# DFHiC: a dilated full convolution model to enhance the resolution of Hi-C data

**DOI:** 10.1093/bioinformatics/btad211

**Published:** 2023-04-21

**Authors:** Bin Wang, Kun Liu, Yaohang Li, Jianxin Wang

**Affiliations:** School of Computer Science and Engineering, Central South University, Changsha 410083, China; Hunan Provincial Key Lab on Bioinformatics, Central South University, Changsha 410083, China; School of Computer Science and Engineering, Central South University, Changsha 410083, China; Hunan Provincial Key Lab on Bioinformatics, Central South University, Changsha 410083, China; Department of Computer Science, Old Dominion University, Norfolk, VA 23529, United States; School of Computer Science and Engineering, Central South University, Changsha 410083, China; Hunan Provincial Key Lab on Bioinformatics, Central South University, Changsha 410083, China

## Abstract

**Motivation:**

Hi-C technology has been the most widely used chromosome conformation capture (3C) experiment that measures the frequency of all paired interactions in the entire genome, which is a powerful tool for studying the 3D structure of the genome. The fineness of the constructed genome structure depends on the resolution of Hi-C data. However, due to the fact that high-resolution Hi-C data require deep sequencing and thus high experimental cost, most available Hi-C data are in low-resolution. Hence, it is essential to enhance the quality of Hi-C data by developing the effective computational methods.

**Results:**

In this work, we propose a novel method, so-called DFHiC, which generates the high-resolution Hi-C matrix from the low-resolution Hi-C matrix in the framework of the dilated convolutional neural network. The dilated convolution is able to effectively explore the global patterns in the overall Hi-C matrix by taking advantage of the information of the Hi-C matrix in a way of the longer genomic distance. Consequently, DFHiC can improve the resolution of the Hi-C matrix reliably and accurately. More importantly, the super-resolution Hi-C data enhanced by DFHiC is more in line with the real high-resolution Hi-C data than those done by the other existing methods, in terms of both chromatin significant interactions and identifying topologically associating domains.

**Availability and implementation:**

https://github.com/BinWangCSU/DFHiC.

## 1 Introduction

In recent years, Hi-C technology (Lieberman-Aiden *et al.* 2009) has become one of the most popular tools in the field of studying 3D genome organization (Schmitt *et al.* 2016). It is based on the traditional chromatin conformation capture (3C) technology, which can study the spatial interaction of chromatin in the whole genome (Van Berkum *et al.* 2010). Similar to other whole-genome sequencing data, Hi-C usually requires millions to billions of paired-end sequencing reads, depending on the genome size and the required resolution (Belton *et al.* 2012). Hi-C interaction analysis promotes the comprehensive recognition of the chromatin organization of the whole genome, which in turn reveals important 3D genome functions (Dixon *et al.* 2012; Wang *et al.* 2016). According to different resolutions of Hi-C data, different structural features can be analyzed (Xiong and Ma 2019), such as loops (Rao *et al.* 2014), topologically associated domains (TADs) (Dixon *et al.* 2012), and A/B compartments (Lieberman-Aiden *et al.* 2009; Fortin and Hansen 2015). However, recognizing finer genome structures requires higher Hi-C resolution, and higher Hi-C resolution requires deeper sequencing depth (Bonev *et al.* 2017), and deeper sequencing depth requires higher experimental cost. Due to the high cost of sequencing, most of the available Hi-C datasets have relatively low-resolution and cannot directly be used to identify finer structures (Zhang *et al.* 2018; Liu *et al.* 2019), such as sub-domains or enhancer–promoter interactions (Jin *et al.* 2013; Dixon *et al.* 2015). Therefore, it is critical to develop an effective computational method, which fully adopts currently available Hi-C data to generate a higher resolution Hi-C interaction matrix for finer structure analysis.

The Hi-C data enhancement task is similar to the image super-resolution task in computer vision (Liu and Wang 2019). In the past few years, to solve the Hi-C data enhancement problem, many deep learning-based data enhancement models have been proposed. Based on the model structure, these methods can be roughly classified into two categories: (i) The Convolutional Neural Network (CNN)-based methods, such as HiCPlus (Zhang *et al.* 2018), SRHiC (Zhang *et al.* 2018), HiCNN (Liu and Wang 2019); (ii) The Generative Adversarial Network (GAN) based methods, such as hicGAN (Liu *et al.* 2019), DeepHiC (Hong *et al.* 2020), HiCSR (Dimmick 2020), VEHiCLE (Highsmith and Cheng 2021), EnHiC (Hu and Ma 2021), and HiCARN (Hicks and Oluwadare 2022).

Among the CNN-based methods, the main focus is on modifying the structure of the method and deepening the model. HiCPlus is the first Hi-C data enhancement model, which is composed of a three-layer CNN. The method learns to map from a low-resolution input to a high-resolution one. SRHiC is based on ResNet (He *et al.* 2016), which improves the Res-block in ResNet by using a smaller convolution kernel to reduce the overall amount of computation. To further improve the enhancement performance, HiCNN employs a highly deep CNN (i.e. 54-layer), where most of the layers use global and local residual connections at the same time.

The GAN-based methods consist of two components: a generator and a discriminator, which are trained in competition with each other. Consequently, the generator can produce the super-resolution Hi-C matrices that are indistinguishable by the discriminator. Early GAN-based methods mainly focused on modifying the loss function. hicGAN is the first GAN-based methods to enhance the Hi-C matrix, and exploits the GAN model to enhance the low-resolution Hi-C matrix into a super-resolution Hi-C matrix. In order to reduce the unclear texture details caused by smoothing, DeepHiC adopts a set of hybrid loss functions: Mean squared error (MSE) loss, total variation loss, perceptual loss, and adversarial loss. In order to further improve the performance, HiCSR incorporates a deep autoencoder to obtain the feature loss function, then adds mean absolute error (MAE) loss and adversarial loss as the final loss function. VEHiCLE also uses a deep autoencoder and incorporates a biologically explicit loss function based on the identification of topologically associated domains to ensure accurate downstream genomic analysis. Recent GAN-based research methods no longer only focus on the impact of loss functions. EnHiC proposes a new convolutional layer (decomposition and reconstruction block) that enables the generator to extract rank-1 matrix features from low-resolution Hi-C matrix at multiple scales and to predict high-resolution Hi-C matrix through a series of subpixel convolutional layers. HiCARN employs a lightweight cascaded convolutional network as generator to achieve higher performance in biological reproducibility and consistency scores.

Currently, DeepHiC outperforms HiCPlus, VEHiCLE, and HiCSR in terms of overall structural similarity scores (Hong *et al.* 2020; Highsmith and Cheng 2021). Moreover, HiCSR, DeepHiC, VEHiCLE in terms of biological reproducibility and consistency scores (GenomeDISCO, HiC-Spector, HiCRep, and QuASAR-Rep) have comparable performance (Highsmith and Cheng 2021). HiCARN yields better GenomeDISCO score than HiCSR and DeepHiC. While each Hi-C enhancement method has its own advantages, further performance improvement is desired in Hi-C applications of exploring genome structures (Hicks and Oluwadare 2022).

One of the main challenges of Hi-C data resolution enhancement is to clearly enhance the high-frequency information details of Hi-C data. The Hi-C data enhanced by existing enhancement methods is still blurrier than high-resolution Hi-C data, which generate a large number of false significant interactions and bias the TAD boundary detection. In addition, the challenge of Hi-C data resolution enhancement is to extract the global pattern of the whole Hi-C matrix. Most of these CNN-based methods rely on a cutting matrix to cut the original Hi-C matrix into parts for resolution enhancement. The usage of cutting matrix has limitation to capture the overall global patterns, particularly the genomic information at the edges of the cutting portion of the original Hi-C matrix. In this paper, we develop a novel dilated fully convolutional network model (DFHiC) to enhance the resolution of Hi-C data. With the help of dilated convolution, DFHiC can utilize peripheral genomic information more efficiently. It effectively enhances the high-frequency information details of Hi-C data, which is helpful for the recovery of significant interactions and the detection of TAD boundaries. In addition, DFHiC has no restrictions on the input size of Hi-C data. More specifically, the limitation effect caused by cutting matrix is eliminated and the Hi-C matrix no longer needs to be divided into several parts to enhance the entire chromosome. Our results indicate that DFHiC enhances low-resolution Hi-C data more effectively than the existing methods, with better scores than other methods in terms of structural similarity (SSIM) and Pearson correlation. More importantly, it outperforms the existing methods in identifying TADs and significant interactions.

## 2 Materials and methods

### 2.1 Materials

We download a high-resolution dataset of four different human cell types (GM12878, K562, IMR90, and NHEK) and a mouse cell type (CH12-LX) from the GEO database, with the accession number GSE63525 (Rao *et al.* 2014). Among them, we use human cell type GM12878 for training, and other cell types are used as test sets to verify the performance of the model. To eliminate the sex effects, we delete the chrX and chrY chromosomes in all human cell types and only retain chromosomes 1–22 (Liu *et al.* 2019). For human GM12878 cell, there are differences between the maternal and paternal chrX. The paternal ChrX is inactive. The inactive ChrX is missing the TAD domains and forms the megadomain and the ultradistal superloops.(Nora *et al.* 2012; Rao *et al.* 2014). These are some structural phenomena that are not present in autosomes. Similarly, we only retain chromosomes 1–19 in the mouse cell type CH12-LX. Afterwards, we merge all aligned Hi-C reads, use the Juicer tool (Durand *et al.* 2016) to generate HIC files, and extract the original chromatin contacts to construct the Hi-C matrices.

When training DFHiC, both the training and testing data come from GM12878. We use the raw reads as the high-resolution Hi-C matrix ground truth. To simulate the low-resolution Hi-C data, we randomly down-sample the raw reads as the low-resolution data. Then, we adopt low-resolution samples with different down-sampling rates ranging from 1/8 to 1/100 to evaluate the performance of the model. The main sampling rate is 1/16, and other ratios are used to evaluate the performance of the model in the cases of different down-sampling rates. To facilitate model training, the processed high-resolution and low-resolution Hi-C matrices are divided into multiple small blocks of the same size without overlapping. Each small block is 40×40 with a resolution of 10 kb. We use the Hi-C matrices extracted from chromosomes 1–17 as the training set. In addition, similar to other Hi-C super-resolution methods, considering the fact that the average genomic distance of TAD is usually <1 Mb, small fragments with a genomic distance <2 Mb between two loci are retained. We use chromosomes 18–22 as the test set with small fragments within 2 Mb genome distance between two loci. The details of the dataset have been listed on Supplementary Table S1. In the case of sufficient memory DFHiC has no required limit on the input size of the data, so DFHiC does not split the Hi-C matrix in the enhancement of the whole chromosome Hi-C matrix.

### 2.2 Methods

**Figure 1.**
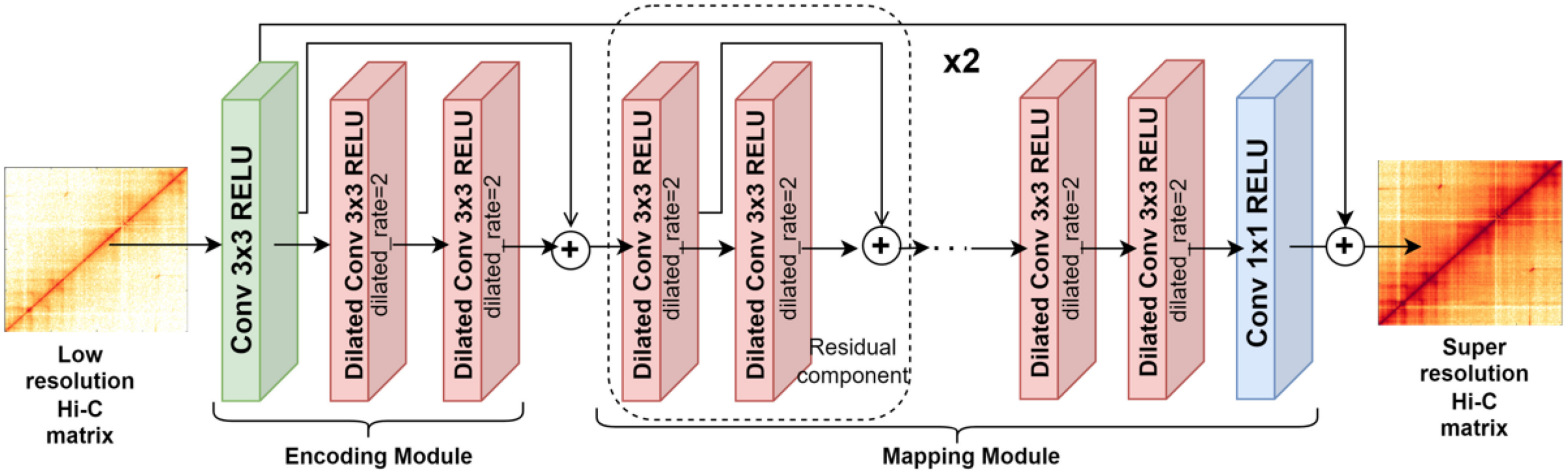
The architecture of DFHiC model. DFHiC consists of vanilla convolution and dilated convolution. It can be divided into Encoding module and Mapping module. The low resolution Hi-C matrix as the input of DFHiC, and the super resolution Hi-C matrix as the output of DFHiC.

DFHiC is a novel fully convolutional network model (Long *et al.* 2015), which is designed to be flexible in handling resolution enhancement of Hi-C matrices of any given size. Figure 1 shows the architecture of DFHiC, which is composed of 10 layers, including vanilla convolution layers and dilated convolution layers, with Rectification Linear Units (RELU) as the activation functions. All convolutional layers incorporate padding operations to ensure that the input and output have the same sizes.

DFHiC extracts effective features to map from the low-resolution matrix to the high-resolution matrix. Specifically, DFHiC includes two modules: the encoding module extracts matrix features and the mapping module converts the low-resolution representations into the high-resolution representations. Here, we describe the model structure of DFHiC in detail:

- **Encoding module:** The encoding module consists of a 3×3 vanilla convolution layer and two 3×3 dilated convolution layers with the dilation rate of 2. The vanilla convolutional layer calculates the local correlations between bins in the low-resolution matrix, while the dilated convolutional layers obtain correlations in further genome distance at a higher semantic level by employing the dilated convolution to increase the receptive field. The correlations are represented as local and global features. The residual connection is used to obtain multi-scale distance information and hierarchical information. Consequently, the output of the encoding module becomes:
  (1) $$F_{e}=C_{e}(L)+D(D(C_{e}(L))),$$
  where $F_{e}$ is the feature extracted by the module, *L* is the input Hi-C matrix, $C_{e}$ is the vanilla convolutional layer with a 3×3 convolution kernel, and *D* is the dilated convolutional layer.
- **Mapping module:** The mapping module consists of two residual components, two 3×3 dilated convolutional layers with the dilation rate of 2 and a 1×1 vanilla convolution. Each residual component is composed of two dilated convolutions connected by residuals. Mapping low-resolution features to high-resolution Hi-C features is obtained by
  (2) $$F_{m}=C_{m}(D(D(R(R(F_{e})))))$$
  (3) $$R(F_{e})=D(F_{e})+D(D(F_{e})),$$

where $F_{m}$ is high-resolution Hi-C features, *R* is the residual component, and $C_{m}$ is a vanilla convolution layer with a 1×1 convolution kernel.

The merge layer serves as the output layer, which outputs the enhanced Hi-C matrix such that where *S* is the enhanced Hi-C matrix finally generated.


(4) $$S=F_{m}+L,$$

Considering *L* as the low-resolution Hi-C matrix, *H* as the high-resolution Hi-C matrix and DFHiC(L) is the enhanced Hi-C matrix obtained by DFHiC for L, the loss function of DFHiC becomes:


(5) $$\begin{array}{c} \frac{1}{n}\sum_{i,j}\left|H_{ij}-\mathrm{DFHiC}{\left(L\right)}_{ij}\right|,n=i*j \end{array}$$

We build our model using the TensorFlow (Abadi *et al.* 2016) and TensorLayer package (Dong *et al.* 2017). The dilation rates are determined by grid search. We trained models with four different dilation rates (2, 5, 7, 9) and determined the dilation rate with the best performance as the final parameter. We adopt a batch size of 128, the epoch of 500, and a learning rate of 0.0001 in an Adam optimizer (Kingma and Ba 2014). The number of channels in each convolution layer of DFHiC is [32, 32, 32, 64, 64, 128, 128, 256, 256, 1], the numbers of channels are the parameters of CNN. During the training process, we divide the training set into training data and validation data according to a certain ratio (9.5:0.5). During training, the training weights of the model are saved according to the optimal MAE results on the validation data. Our model is trained on GPU 2080 Ti.

### 2.3 Evaluation metrics

We evaluate the quality of samples generated by our model from multiple perspectives. First, we use MSE, which calculates the average square error between the enhanced super-resolution Hi-C matrix and the actual high-resolution Hi-C matrix based on the genomic distance measure. MSE can reflect the average difference of each position of the Hi-C matrix. Then, we also compare the peak signal-to-noise ratio (PSNR) (Huynh-Thu and Ghanbari 2008) and structural similarity index (SSIM) (Wang *et al.* 2004) between the two Hi-C matrices, which are commonly used evaluation indicators for evaluating image super-resolution in the field of computer vision. where the Hi-C matrix is n×n, $H_{ij}$ is the pixel in the real high-resolution Hi-C matrix, $S_{ij}$ is the pixel in the enhanced Hi-C matrix, and *MAX* is the largest value in *H*. SSIM is an overall measure that also considers the contrast and structural similarity of the two comparison images. where $\mu_{H}$ is the mean of *H*, $\mu_{S}$ is the mean of *S*, $\sigma_{H}$ is the variance of *H*, $\sigma_{S}$ is the variance of *S*, $\sigma_{HS}$ is the covariance of *H* and *S*, *R* is the dynamic range of the pixel values, $c_{1}=0.01$ and $c_{2}=0.03$.


(6) $$\begin{array}{c} \mathrm{MSE}=\frac{1}{n^{2}}\sum_{i,j}{\left(H_{ij}-S_{ij}\right)}^{2}\\ \mathrm{PSNR}=10{\;\text{log}\;}_{10}(\frac{MAX^{2}}{\mathrm{MSE}}), \end{array}$$

(7) $$\mathrm{SSIM}=\frac{\left(2\mu_{H}\mu_{S}+{\left(c_{1}*R\right)}^{2})(2\sigma_{HS}+{\left(c_{2}*R\right)}^{2}\right)}{\left(\mu_{H}^{2}+\mu_{S}^{2}+{\left(c_{1}*R\right)}^{2})(\sigma_{H}^{2}+\sigma_{S}^{2}+{\left(c_{2}*R\right)}^{2}\right)},$$

In addition, we use the Pearson correlation coefficient and Spearman rank correlation coefficient. Both measure the correlation between the predicted Hi-C matrix and the actual Hi-C matrix, which also depends on the genomic distance. Here, we group interactions with the same genomic distance and calculated Pearson’s correlation coefficient and Spearman rank correlation coefficient between the real interactions and interactions predicted by different methods after grouping.

## 3 Result analysis

### 3.1 Reconstruction of super-resolution Hi-C matrix from low-resolution Hi-C matrix

DFHiC is trained on chromosomes 1–17 of the GM12878 cell type and is tested and evaluated on chromosomes 18–22. In addition, to compare with DeepHiC and HiCARN, we use the training weights provided from the official ones, and the data are normalized according to the data preprocessing method in the DeepHiC method. For comparison, we denormalize the results obtained by the DeepHiC and HiCARN methods. To fully evaluate the performance of the model, we calculate multiple evaluation indicators to compare the low-resolution Hi-C matrix enhanced by all enhancement methods. Table 1 shows the MSE, PSNR, and SSIM scores of all enhanced methods in the test set compared to high-resolution. As shown in Table 1, DFHiC scores significantly better than the compared enhancement methods in the cases of these three metrics. In addition, we normalize the label and the results of other methods by the data preprocessing of DeepHiC and HiCARN. Then we calculate the scores using these three metrics. The details can be seen in Supplementary Table S2.

**Table 1.** The comparison results of the enhancement methods on the test set.

Method	MSE	PSNR	SSIM
HiCPlus	22.882	19.388	0.275
SRHiC	22.056	19.391	0.209
HiCNN	16.884	20.554	0.295
DeepHiC	16.986	19.895	0.273
HiCARN	15.760	20.104	0.276
**DFHiC**	**14.201**	**21.133**	**0.342**

Note: The top scores and methods are indicated in bolded.

Here, we calculate the three metric scores with respect to different genomic distances for further analysis. As shown in Supplementary Table S3, as the genomic distance increases, the metric scores of all enhancement methods are getting lower. This is because the further away from the diagonal in the Hi-C matrix, the sparser the matrix with less information, resulting in gradually lower scores. In addition, compared with other methods, DFHiC has significant improvement on all genome distances. Supplementary Fig. S1 shows the performance of all methods in terms of PSNR and SSIM at different genomic distances.

To quantitatively evaluate the model performance, we also calculate the Pearson correlation coefficient and the Spearman rank correlation coefficient between these enhancement methods and the high-resolution matrix. Supplementary Fig. S2 shows the scores of Pearson correlation coefficient and Spearman correlation coefficient for all enhancement methods in the test set. One can observe that, compared with the low-resolution Hi-C matrix, the enhanced Hi-C matrix obtained by all enhancement methods significantly improves the correlation coefficient scores and furthermore, DFHiC has a more prominent improvement, particularly in the Spearman correlation coefficient score.

All enhancement methods are trained on NVidia TITAN Xp GPU. The time and memory cost for each method to enhance a Hi-C data of size 40×40 are shown in Supplementary Table S4. We observe that DFHiC outperforms HiCARN in terms of training time and memory required for per epoch. DFHiC achieves a shallower network architecture than the lightweight HiCARN with computational performance improvement.

In order to evaluate the effectiveness of the dilated convolution and the choice of the dilation rate in DFHiC, we do ablation experiments for comparing the vanilla convolution with the dilated convolution. In this experiment, we replace all dilated convolution layers in DFHiC with vanilla convolution layers while keeping the number of layers unchanged. The effect of different dilation rates on the model performance is also compared. The detailed comparison results can be found in Supplementary Table S5 and Supplementary Fig. S3. We observe that the dilated convolution has a better performance than the vanilla convolution. Furthermore, regarding the dilated convolution, the performance decreases gradually as the dilated ratio increases. Dilated convolution can help the model to obtain higher performance when the genomic distance is 0. We compare the time required for per epoch when training with the dilated convolution and the vanilla convolution. One can observe that dilated convolution is more time consuming than vanilla convolution when training, which can be seen in Supplementary Table S6 for details.

DFHiC adopts MAE as its loss function instead of MSE in most of the other enhancement methods. This is because DFHiC can obtain better performance when training DFHiC using MAE as the loss function. The specific comparison results can be found in Supplementary Table S7.

### 3.2 Evaluation of DFHiC based on its ability to recover significant interactions

**Figure 2.**
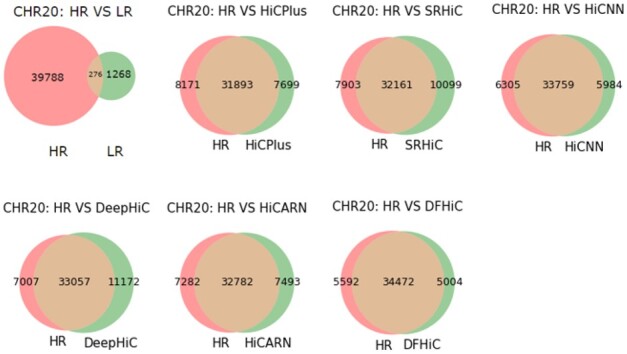
The number of significant interactions detected by Fit-Hi-C on chromosome 20 recovered by different methods. HR is high-resolution Hi-C data. The red part represents the number of significant interactions in the high-resolution Hi-C data that have not been recovered by the enhancement method, the orange part is the number of the significant interactions recovered by the enhancement method, and the green part includes the significant interactions not in the high-resolution Hi-C data generated by the enhancement method.

To evaluate DFHiC contribution to the recognition of chromatin contacts and chromatin loops, we use the Fit-Hi-C tool (Ay *et al.* 2014) to identify chromatin interactions by estimating the statistical confidence of intrachromosomal interactions. The Fit-Hi-C tool is commonly used to identify significant interactions in high-resolution Hi-C data and enhanced Hi-C data. We called significant interactions with a strict threshold (*q*-value <1e−6). Then, we only retained significant interactions with genomic distances in the range of 30–300 kb. We use chromosomes 18–22 of the GM12878 cell type for evaluation. We apply the Fit-Hi-C tool to the high-resolution Hi-C matrix, the low-resolution Hi-C matrix obtained by down-sampling, and the super-resolution Hi-C matrix obtained by various enhancement methods. Figure 2 shows the number of significant interactions detected by Fit-Hi-C, and the intersection parts represent the same number of reused significant interactions. The results on other chromosomes are also shown in Supplementary Fig. S4. One can observe that Fit-Hi-C detects only a small number of significant interactions from the low-resolution Hi-C data. All enhancement methods do enhance the low-resolution Hi-C data, obtaining more significant interactions consistent with the high-resolution Hi-C data. Compared to other enhancement methods, DFHiC can recover more significant interactions while reducing pseudo-significant interactions.

Since all enhancement methods produce a different number of significant interactions of chromosomes, it is insufficient to compare the number of significant interactions that recover the original high-resolution Hi-C matrix. We evaluate all enhancement methods via the F1 score, which is the harmonic mean of precision and recall. The F1 scores of all methods to recover significant interactions of chromosomes are listed in Supplementary Table S8. DFHiC shows better performance than other enhancement methods on chromosomes 18–22. In addition, we calculate the false positive rate of all methods, which can be seen in Supplementary Table S9. The false positive rate of DFHiC is range from 12.7% to 16.5%. The average false positive rate of DFHiC on chromosomes 18–22 is only 13.9%, which is much lower than the average false positive rate of other methods. To this end, we plot the Receiver Operating Characteristic curves and calculate the AUC score, which can be seen in Supplementary Fig. S5 for details. We observe that the AUC score of DFHiC method is higher than that of other enhancement methods on chromosomes 18–22.

To further analyze the recovery of significant interactions, we do the visual analysis of significant interactions on chromosome 20 of GM12878 in the region 10–11 Mb, which can be seen in Supplementary Fig. S6. The false positive significant interactions obtained by all enhancement methods appear mainly in the neighborhood of true positive significant interactions.

In addition, we count the *P*-value of significant interactions for further analysis. The mean values of *P*-value for true positive, false positive and false negative significant interactions recovered by all enhancement methods are presented in supplementary Tables S10–S12, respectively. We observe that the mean values of *P*-value for true positive significant interactions recover by all enhancement methods are mainly concentrated in 1e−9, while the mean values of *P*-value for false positive and false negative significant interactions for all enhancement methods are significantly concentrated in 1e−8. The mean values of *P*-value for the false positive and false negative significant interactions are higher than the mean values of *P*-value for the true positive significant interactions.

### 3.3 Evaluation of DFHiC based on its ability to identify cell type-specific contact domain boundaries

A lot of analysis in the previous studies have been conducted in Hi-C data and find that there are conserved spatial structures in the genome, among which Topologically Associating Domains (TADs) are one of the widely reported spatial structures. Therefore, we use the Arrowhead (Rao *et al.* 2014) method to perform TAD detection on low-resolution Hi-C data, high-resolution Hi-C data, and Hi-C data enhanced by enhancement methods, and the Jaccard Index metric is calculated for the set of TAD boundaries of high-resolution Hi-C data and Hi-C data from other enhancement methods. Here, we compare chromosomes 18–22 in GM12878, and report the results in Table 2.

**Table 2.** Comparison of TAD boundary detected by the enhancement methods on chromosomes 18–22 in GM12878.

Method	Chr18	Chr19	Chr20	Chr21	Chr22	Avg
LR	0.026	0.159	0.094	0.101	0.166	0.109
HiCPlus	0.377	0.508	0.505	0.46	0.551	0.48
SRHiC	0.429	0.486	0.453	0.403	0.541	0.462
HiCNN	0.527	0.6	0.587	0.568	0.59	0.574
DeepHiC	0.502	0.517	0.495	0.532	0.488	0.507
HiCARN	0.527	0.546	0.55	0.489	0.566	0.536
**DFHiC**	**0.546**	**0.616**	**0.626**	**0.647**	**0.678**	**0.623**

Note: The top scores and methods are indicated by bolded.

**Figure 3.**
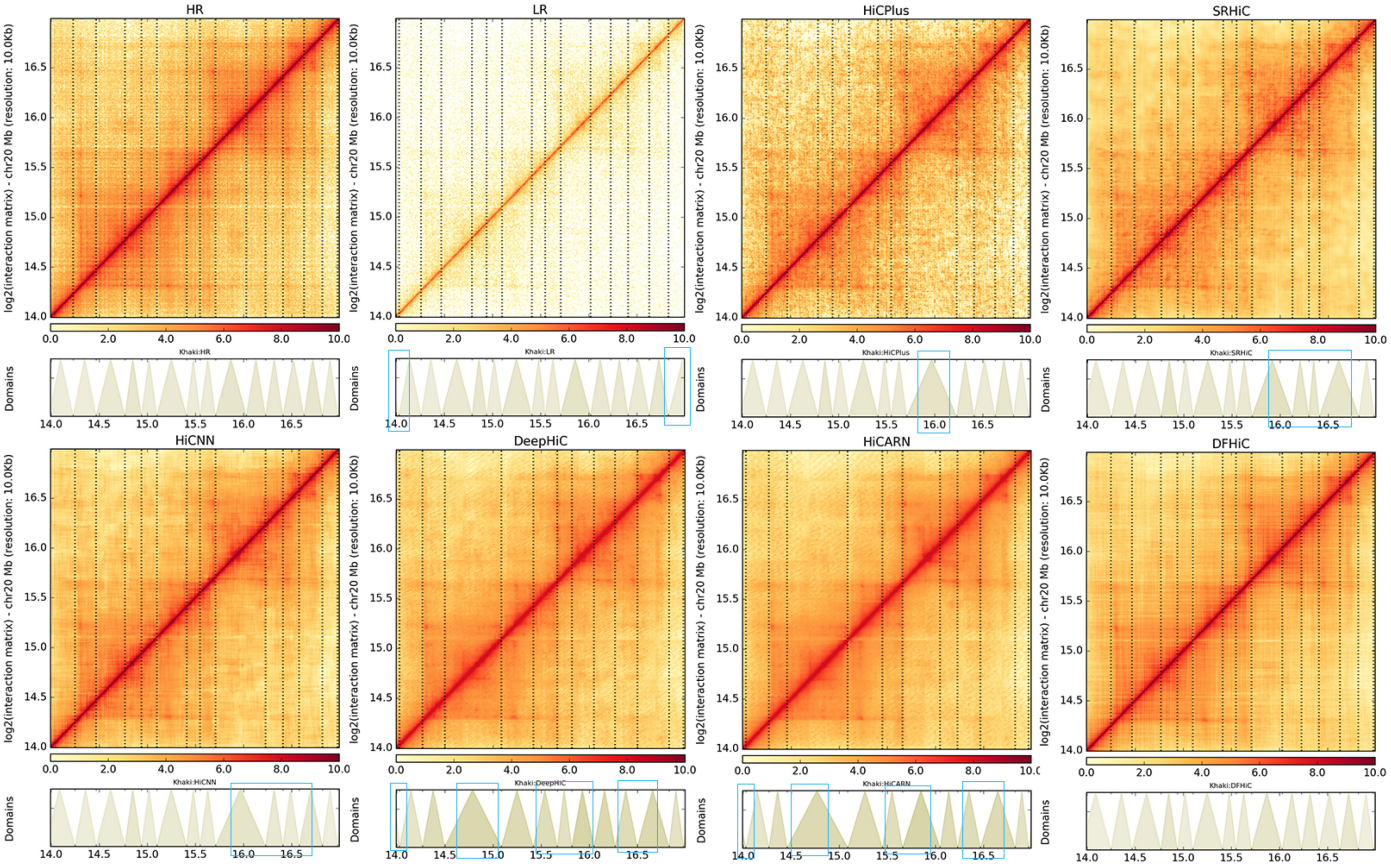
Visualization of Hi-C data and detected TAD on chr20 (14–17 Mb) by HiCPlotter. LR is low-resolution Hi-C data and HR is high-resolution Hi-C data. The 14–17 Mb region on chromosome 20 and the TAD corresponding to this region are visualized. “Domain” is the visualization of the detected TAD. The green box marks the part where the TAD detection result deviates from the high-resolution Hi-C matrix.

We use the HiCPlotter (Akdemir and Chin 2015) tool to visualize the low-resolution Hi-C matrix, the high-resolution Hi-C matrix, and the super-resolution matrix reconstructed from DFHiC, and identify the contact domain boundaries of the corresponding cell types. We extract a 3 Mb genomic region (Chr20: 14–17 Mb) for evaluation. Figure 3 shows the enhanced Hi-C matrix detected TAD. In detecting the TAD areas, compared with the low-resolution Hi-C matrix, the Hi-C matrices enhanced by the enhancement methods all exhibit certain enhancements in recovery. The differences between the enhanced Hi-C matrix and the high-resolution Hi-C matrix are highlighted with blue boxes. More details are shown in Supplementary Fig. S7.

### 3.4 Evaluation of DFHiC based on its ability to reproduce high-resolution Hi-C matrices

Taking into account the unique characteristics of the Hi-C matrix, such as sequencing depth, distance dependence, and domain structure, we evaluate the biological reproducibility of the two matrices by adopting the GenomeDISCO (Ursu *et al.* 2018), HiCRep (Yang *et al.* 2017), HiC-Spector (Yan *et al.* 2017) and QuASAR-Rep (Sauria *et al.* 2015) tools to calculate the scores. We use 3DChromatin_ReplicateQC (Yardımcı *et al.* 2019), which is composed of these four repeatability measures. The GenomeDISCO tool, which is a commonly used comparison method, smooths the data before comparison and then calculates the reproducibility score via a random walk method. QuASAR-Rep is a part of the hifive suite, which is a set of tools to handle Hi-C and 5C data. We calculate the reproducibility scores of the Hi-C matrix obtained by all enhancement methods and the high-resolution Hi-C matrix for the entire chromosome 18–22. We report the results with regard to GenomeDISCO and QuASAR-Rep in Table 3 and HiCRep and HiC-Spector in Supplementary Table S13.

**Table 3.** The biological reproducibility scores of all enhanced Hi-C matrices and high-resolution Hi-C matrices on chromosomes 18–22 in GM12878.

	Method	Chr18	Chr19	Chr20	Chr21	Chr22	Avg
**Genome DISCO**	HiCPlus	0.802	0.837	0.819	0.832	0.861	0.83
	SRHiC	0.479	0.767	0.677	0.507	0.511	0.588
	HiCNN	0.749	0.805	0.772	0.794	0.847	0.793
	DeepHiC	0.438	0.43	0.418	0.124	0.108	0.304
	HiCARN	0.708	0.826	0.814	0.787	0.788	0.785
	**DFHiC**	**0.835**	**0.906**	**0.888**	**0.888**	**0.923**	**0.888**
**QuASAR-Rep**	HiCPlus	0.899	0.95	0.928	0.917	0.95	0.929
	**HiCNN**	0.936	0.969	0.959	0.942	0.97	**0.955**
	SRHiC	0.921	0.962	0.951	0.924	0.96	0.944
	DeepHiC	**0.939**	0.954	0.952	**0.945**	0.957	0.949
	HiCARN	**0.939**	0.958	0.952	0.944	0.96	0.95
	**DFHiC**	0.934	**0.97**	**0.96**	0.94	**0.972**	**0.955**

Note: The top scores and methods are indicated by bolded.

One can observe from Table 3 that the Hi-C matrix enhanced by DFHiC has achieved high scores in terms of reproducibility. By averaging the results of the testing chromosomes, DFHiC obtains the GenomeDISCO’s score of 0.888, which clearly surpass the compared methods.

### 3.5 Resolution enhancement of actual low-resolution Hi-C data

We perform data enhancement on the actual low-resolution Hi-C data. The actual low-resolution data come from GEO GSM1551550 (HIC001) of GM12878. The high-resolution data from GEO database (GSE63525) is constructed by combining *in situ* Hi-C samples obtained from multiple independent experiments. Therefore, we can recover the Hi-C samples obtained in each independent experiment as the low-resolution Hi-C data generated by the experiment. We use DFHiC trained by GM12878 (down-sampling rate is 1/16) to enhance the actual low-resolution Hi-C data. Here, we enhance the low-resolution chromosomes 18–22 because these chromosomes are not used for model training.

**Figure 4.**
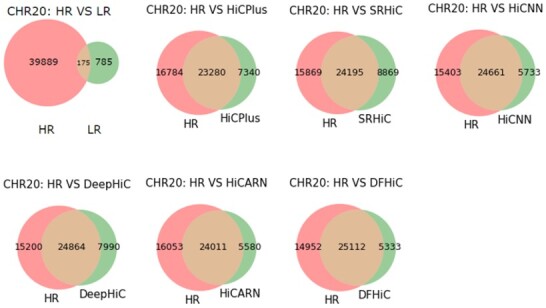
The number of significant interactions detected by Fit-Hi-C on chromosome 20 recovered by different methods for actual low-resolution Hi-C data.

We use the Fit-Hi-C tool to calculate all peaks of all enhanced methods when *q*-value<1e−06. Furthermore, we only retained significant chromatin loops with genomic distances in the range of 30–300 kb. Figure 4 shows the quantity of recovery of high-resolution Hi-C data from the actual low-resolution Hi-C matrix enhanced by all methods on chromosome 20. The results on other chromosomes are shown in Supplementary Fig. S8. The F1 scores of significant interactions of chromosomes recovered by all methods at low-resolution Hi-C data are listed in Supplementary Table S14. One can observe that the Hi-C matrix enhanced by DFHiC has a better ability to recover the significant interactions than the compared methods, measured with fewer pseudo significant interactions generated. Similarly, we calculate the false positive rate of all methods on different chromosomes for enhanced actual low-resolution Hi-C data, and the results are shown in Supplementary Table S15. DFHiC gets lower false positive rate on different chromosomes, its false positive rate ranges from 20.3% to 29.7%. The average false positive rate of DFHiC is only 23.2% on chromosomes 18–22, which is better than the average false positive rate of other methods.

We use the Arrowhead method to perform TAD detection on actual low-resolution Hi-C data, high-resolution Hi-C data, and Hi-C data enhanced by all methods, and calculate Jaccard index metrics for the TAD boundary sets of high-resolution Hi-C data and Hi-C data from other enhancement methods. Here, we compare chromosomes 18–22 of GM12878 and report the results in Supplementary Table S16. The TAD boundaries detected by the DFHiC-enhanced Hi-C data in general are closer to those of the high-resolution Hi-C data. We use the HiCPlotter tool to visualize the actual low-resolution Hi-C data, the high-resolution Hi-C data, and the Hi-C data enhanced by all methods to determine the corresponding contact domain boundaries, which can be seen in Supplementary Fig. S9.

We calculate the reproducibility scores of the Hi-C data obtained by all enhancement methods for the actual low-resolution Hi-C data and the high-resolution Hi-C data on the entire chromosomes 18–22, which can be seen in Supplementary Table S17 for details.

### 3.6 Resolution enhancement between different cell types and different species

We conduct experiments to verify whether the training model can enhance the low-resolution Hi-C data of different cell types (K562, IMR90, NHEK) of the same species. In addition, we use low-resolution mouse cell types (CH12-LX) to verify whether the trained model can enhance Hi-C data of different species. In this experiment, K562, NHEK, IMR90, and CH12-LX have a resolution of 10 kb. They all have a down-sampling rate of 1/16. We use the model trained on GM12878 to directly enhance the low-resolution Hi-C data of other human cell types (K562, IMR90, NHEK) and mouse cell types (CH12-LX). The low-resolution Hi-C data of different cell types are also obtained through random down-sampling. For the testing on human cell types, we use Hi-C data from chromosomes 18–22; for the testing on mouse cell types, we use Hi-C data from chromosomes 16–19. Then, we evaluate all enhancement methods in terms of the MSE, PSNR, and SSIM scores. Finally, we report the results on K562 and IMR90 in Table 4, and on other different cell types in Supplementary Table S18.

**Table 4.** The comparison results of the enhancement methods on the test set from different cell types and different species.

	Method	MSE	PSNR	SSIM
**K562**	HiCPlus	10.568	12.959	0.208
	HiCNN	3.604	17.406	0.184
	SRHiC	3.973	16.85	0.11
	DeepHiC	4.018	17.025	0.189
	HiCARN	3.539	17.739	0.199
	**DFHiC**	**3.254**	**17.933**	**0.237**
**IMR90**	HiCPlus	10.144	13.406	0.212
	HiCNN	3.565	17.626	0.19
	SRHiC	3.95	17.1	0.133
	DeepHiC	3.964	17.257	0.188
	HiCARN	3.485	18.047	0.2
	**DFHiC**	**3.274**	**18.171**	**0.261**

Note: The top scores and methods are indicated by bolded.

In these three metrics, DFHiC achieves significantly better scores than the compared methods, which yields better generalization in different cell types or species. Furthermore, this indicates that DFHiC can effectively learn the mapping relationship from low-resolution Hi-C data to high-resolution ones.

To evaluate DFHiC contribution to the recognition of chromatin contacts and chromatin loops in different cell types, we use the Fit-Hi-C tool to evaluate chromosome 19 enhanced by all methods in different cell types. Again, the threshold for significant interactions is set here to *q*-value <1e−6 to preserve significant interactions. Then, we only retained significant interactions with genomic distances in the range of 30–300 kb. We evaluated the performance using the F1 score as shown in Table 5. Significant interactions recovered on different cell types by DFHiC outperformed other approaches. Supplementary Fig. S10 shows the number of significant interactions detected by Fit-Hi-C, and the intersection parts represent the same number of reused significant interactions. Here, we also calculated the false positive rate of all methods on chromosome 19 for different cell types, and the results are presented in Supplementary Table S19. DFHiC obtains lower false positive rate on different cell types, and its false positive rate ranges from 20.4% to 23.1%, with less volatility than other methods. The average false positive rate of DFHiC in different cell types is only 21.3%, which is much better than the average false positive rate of other methods.

**Table 5.** The comparison results of F1 scores recovered by all methods for significant interactions of chromosome 19 on different cell types.

Method	K562	IMR90	NHEK	CH12-LX
LR	0.001	0.001	0.001	0.004
HiCPlus	0.643	0.66	0.253	0.576
SRHiC	0.567	0.593	0.299	0.508
HiCNN	0.713	0.722	0.388	0.683
DeepHiC	0.653	0.684	0.297	0.611
HiCARN	0.659	0.692	0.346	0.637
**DFHiC**	**0.754**	**0.754**	**0.419**	**0.678**

Note: The top scores and methods are indicated by bolded.

For TAD boundary detection, we also calculate Jaccard index metrics for different cell types (K562, IMR90, NHEK, CH12-LX). Here, we compare different cell types and report the results in Supplementary Table S20 (K562), Supplementary Table S21 (IMR90), Supplementary Table S22 (NHEK) and Supplementary Table S23 (CH12-LX). We observe that the TAD boundaries detected by DFHiC-enhanced Hi-C data in different cell types are closer to the TAD boundaries detected by high-resolution Hi-C. We use the HiCPlotter tool to visualize results for different cell types, as seen in Supplementary Fig. S11 (K562), Supplementary Fig. S12 (IMR90), Supplementary Fig. S13 (NHEK), and Supplementary Fig. S14 (CH12-LX).

We calculate the reproducibility scores of the Hi-C data obtained by all methods for different cell types and the high-resolution Hi-C data on the entire chromosome 18–22, which can be seen in Supplementary Table S24 (K562), Supplementary Table S25 (IMR90), Supplementary Table S26 (NHEK) and Supplementary Table S27 (CH12-LX) for detail.

### 3.7 Enhancement effect in the cases of different down-sampling ratios

We randomly down-sample the high-resolution Hi-C data of GM12878 at different down-sampling rates (1/8, 1/25, 1/50, 1/100) to obtain different low-resolution data. Taking the Hi-C data with a resolution of 10 kb as an example, the data obtained by random sampling at a down-sampling rate of 1/16 theoretically generates the Hi-C data with a resolution of 160 kb, the data obtained by random down-sampling at a sampling rate of 1/25 theoretically produces the Hi-C data with a resolution of 250 kb, and the data obtained by random sampling at a down-sampling rate of 1/100 theoretically generates the Hi-C data with a resolution of 1 Mb. We retrain all methods on the down-sampled datasets. Similarly, we use chromosomes 18–22 as the test set for evaluation and comparison. Here, we calculate three metrics for all methods at different resolutions: MSE, PSNR, and SSIM. Table 6 shows that DFHiC performs much better than the compared methods on different resolution Hi-C data in the cases of 1/8 and 1/25. Furthermore, Supplementary Table S28 show the results in other cases of the down-sampling ratios.

**Table 6.** The comparison results of all enhancement methods on the test set of low-resolution Hi-C samples down-sampled in the cases of different ratios.

	Method	MSE	PSNR	SSIM
**Ratio 1:8**	HiCPlus	20.246	16.958	0.396
	HiCNN	9.624	20.218	0.405
	SRHiC	63.13	12.816	0.348
	DeepHiC	9.756	19.762	0.457
	HiCARN	8.796	20.092	0.459
	**DFHiC**	**8.701**	**20.61**	**0.514**
**Ratio 1:25**	HiCPlus	50.463	13.068	0.198
	HiCNN	12.776	19.04	0.213
	SRHiC	25.937	16.298	0.143
	DeepHiC	13.509	18.767	0.274
	HiCARN	11.685	19.213	0.271
	**DFHiC**	**11.59**	**19.454**	**0.319**

Note: The top scores and methods are indicated in bolded.

One can observe that, as the down-sampling ratio increases, the enhancement effect of all methods tends to be downward. This is due to the fact that more information is lost in the cases of the higher down-sampling ratio of low-resolution Hi-C data. Consequently, the information loss prevents the machine learning model from effectively enhancing the matrix. The SSIM score decreases most significantly, indicating that it is difficult for the model to learn effective structural information when critical information is lost in the low-resolution Hi-C data.

To further analyze the model to be able to enhance the Hi-C matrix with higher resolution, we train DFHiC on the Hi-C data with a resolution of 50 kb. Then we use DFHiC to enhance the Hi-C data with a 50 kb resolution to Hi-C data with a 5 kb resolution. We use HiCPlotter to visualize the results on chromosome 20 (14–15.5 Mb), as seen in Supplementary Fig. S15. We observe that the Hi-C data enhanced by DFHiC is closer to the high-resolution Hi-C data in terms of boundary detection of TAD.

## 4 Conclusion

In this paper, we develop a dilated full convolution model to effectively and conveniently enhance the resolution of Hi-C data. We verify the quality of the Hi-C matrix reconstructed by DFHiC in some scenarios. The extensive experimental results show that DFHiC is superior to other methods under different measures. The Hi-C matrix reconstructed by DFHiC is highly similar to the high-resolution Hi-C matrix, which allows meaningful interactions to be detected from the low-resolution Hi-C matrix at a low cost. This also enables the finer structure (e.g. TADs, loop) in the chromosome to be detected.

We also observe that there are some commonalities in the local patterns between different cell types of the same species. As a result, DFHiC can still achieve better scores when trained in other cell types. However, due to the differences among species, local characteristic information cannot be used to better enhance the Hi-C data across different species.

## Supplementary Material

btad211_Supplementary_Data

## Data Availability

The data underlying this article are available from the GEO database, with the accession number GSE63525, at https://www.ncbi.nlm.nih.gov/geo/query/acc.cgi?acc=GSE63525.
