# Supplementary material for: DFHiC: a dilated full convolution model to enhance the resolution of Hi-C data
Source: Bioinformatics. 2023 Apr 21;39(5):btad211. doi: 10.1093/bioinformatics/btad211 (PMC10166584; doi:10.1093/bioinformatics/btad211)
Supplement: btad211_Supplementary_Data [file btad211_supplementary_data.docx]

**Supplementary Materials**

# Supplementary Figures


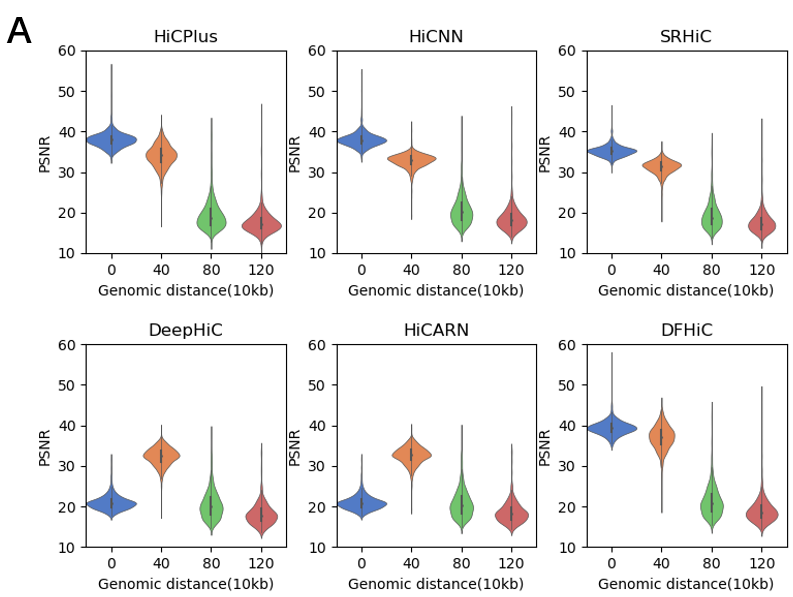


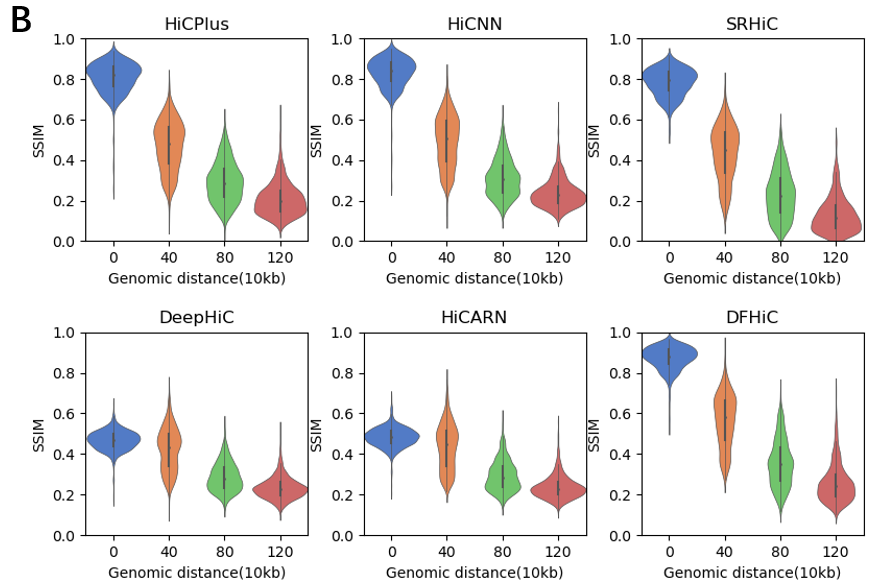


**Fig. S1. Comparison of the PSNR and SSIM results of the all enhancement methods on the test set at different genomic distances. (A)** The violin plots of PSNR for all enhancement methods at different genomic distances (genomic distances of 0, 400kb, 800kb, 1200kb). **(B)** The violin plots of SSIM for all enhancement methods at different genomic distances (genomic distances of 0, 400kb, 800kb, 1200kb).


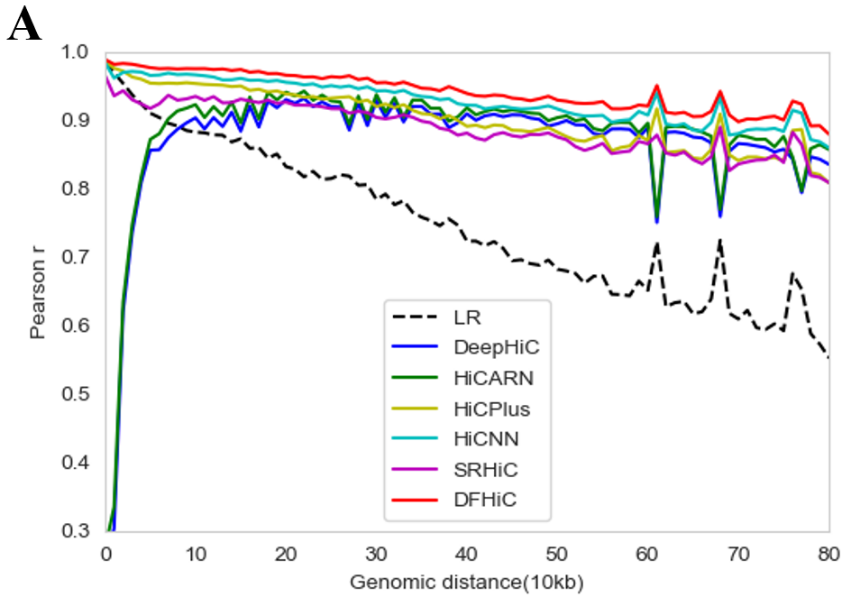


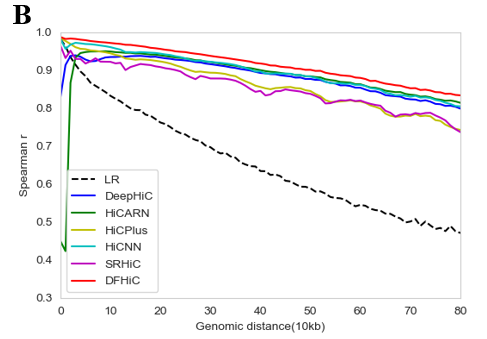


**Fig. S2. Spearman rank correlation coefficient scores and Pearson correlation coefficient scores of all enhancement methods in the test dataset. (A)** The comparison results of Pearson correlation of all methods at different genomic distances. **(B)** The comparison results of Spearman correlation of all methods at different genomic distances.

**
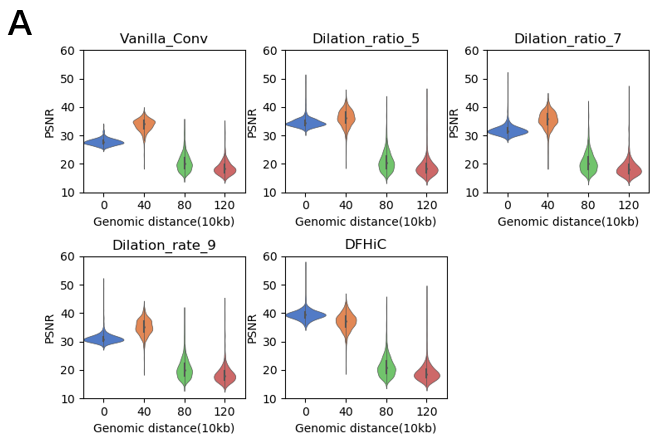
**

**
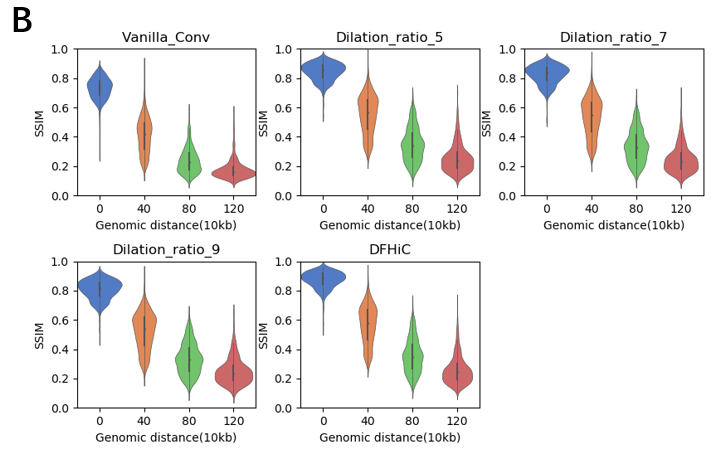
**

**Fig. S3. Comparison of the PSNR and SSIM results of the ordinary convolution and dilation rate on the test set at different genomic distances. (A)** The violin plots of PSNR for the ordinary convolution and dilation rate at different genomic distances (genomic distances of 0, 400kb, 800kb, 1200kb). **(B)** The violin plots of SSIM for the ordinary convolution and dilation rate at different genomic distances (genomic distances of 0, 400kb, 800kb, 1200kb).

**
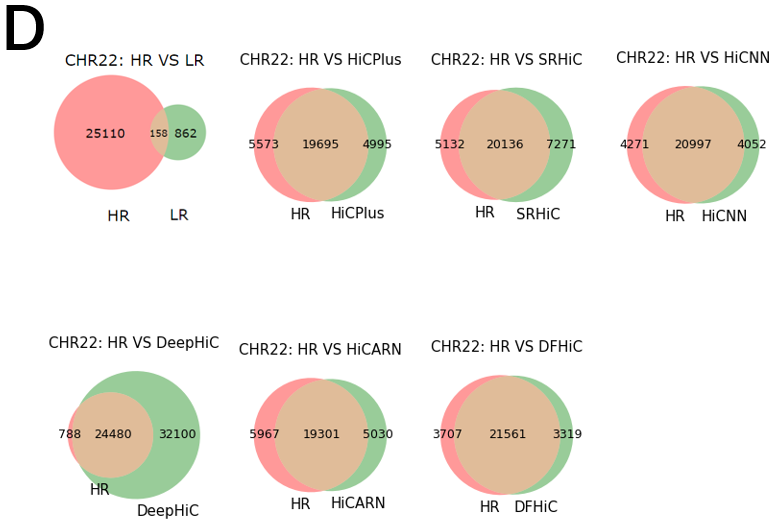
**
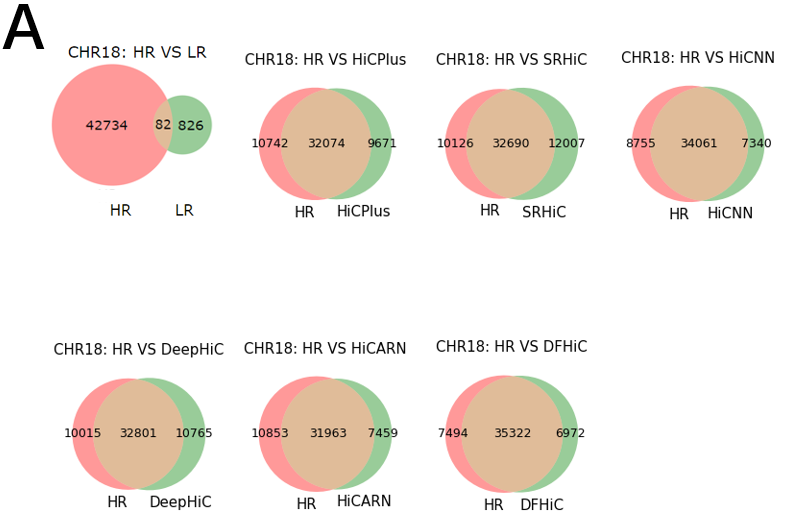


**
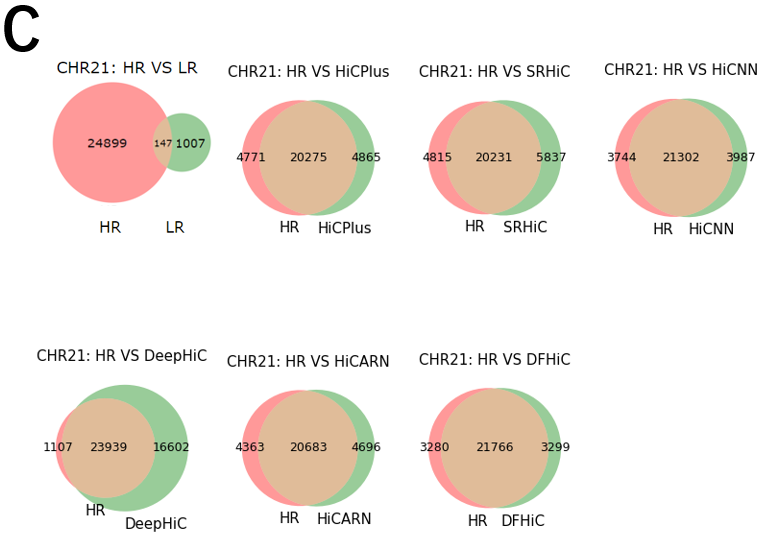
**
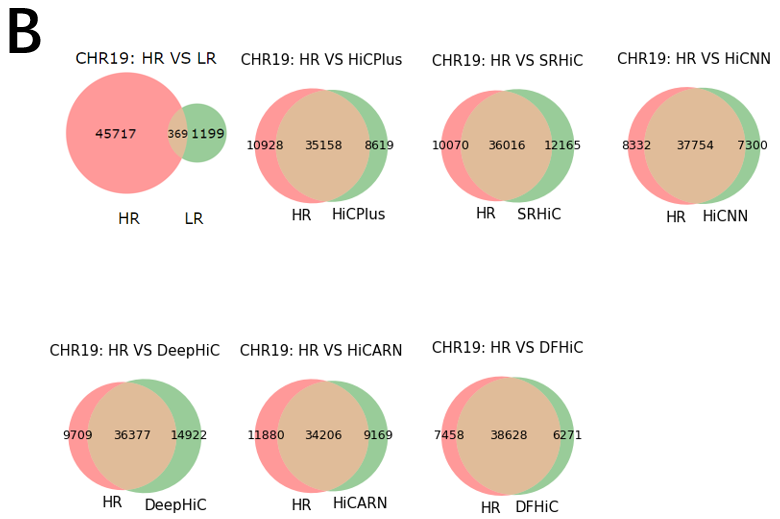


**Fig. S4. The number of significant interactions detected by Fit-Hi-C on different chromosomes recovered by different methods. (A**) Chromosome 18. **(B**) Chromosome 19. **(C)** Chromosome 21. **(D)** Chromosome 22.


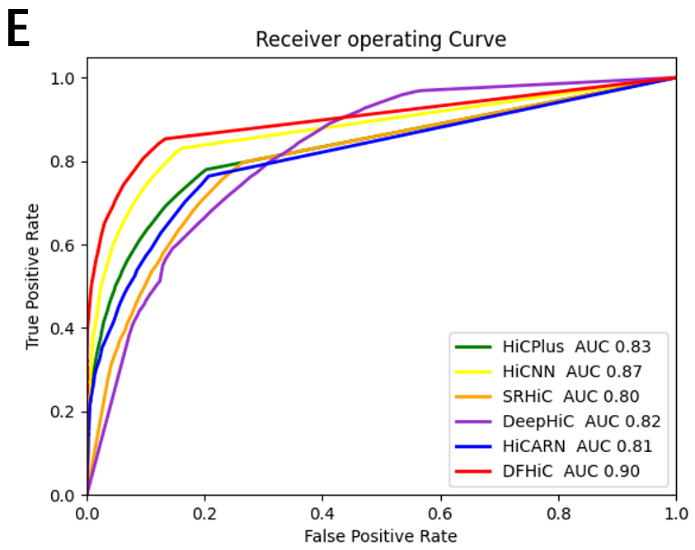
**
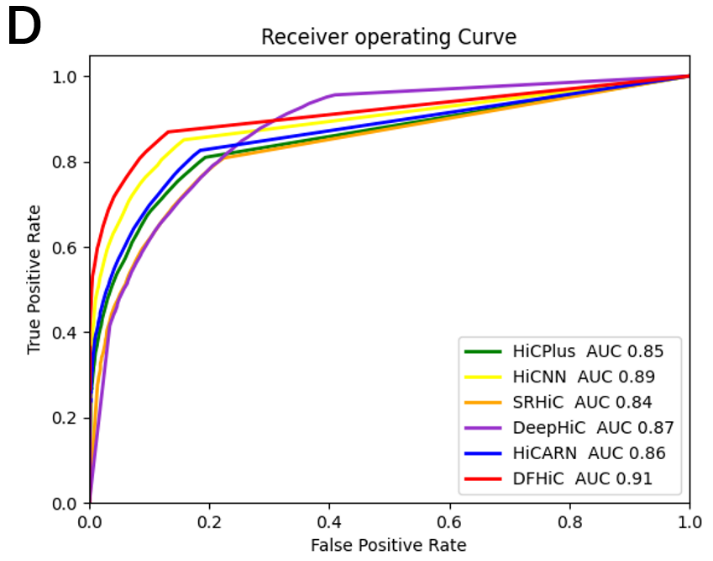

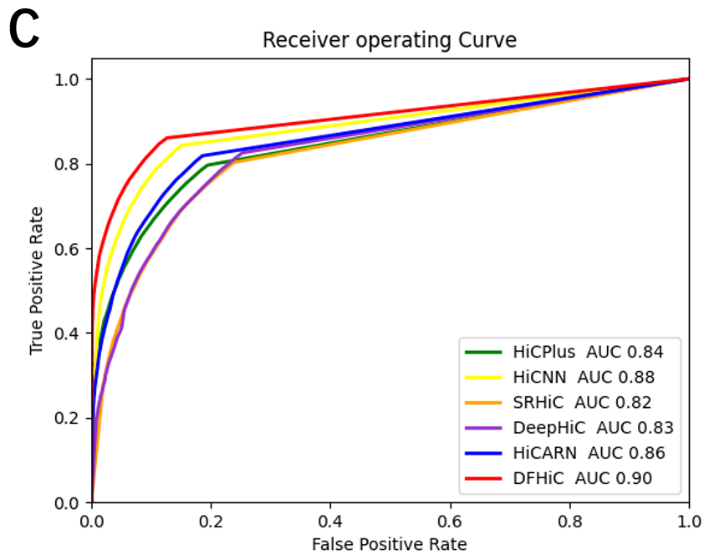
**
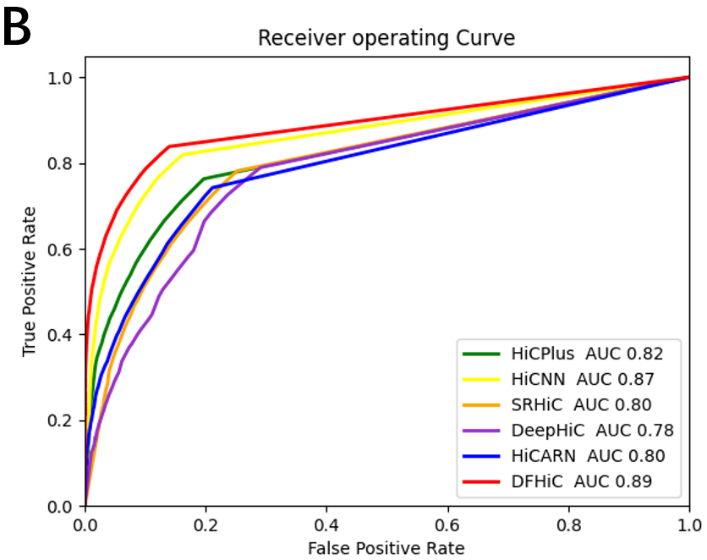

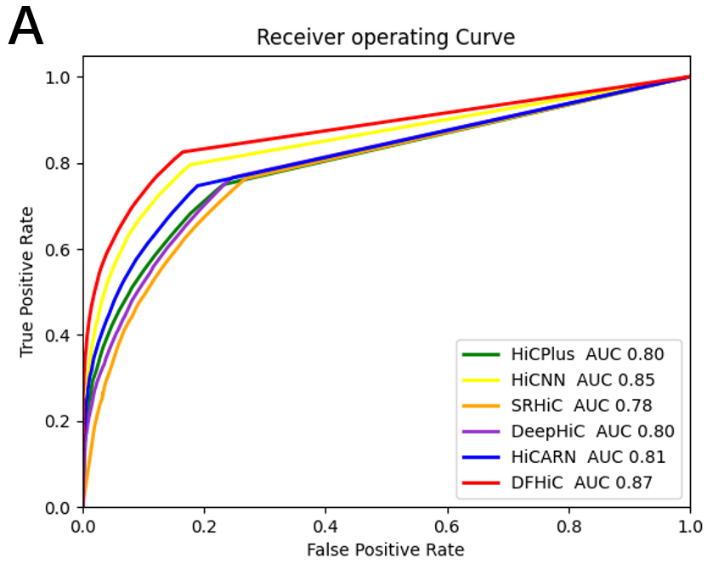
**Fig. S5.** **The Receiver Operating Characteristic (ROC) curves of all enhancement methods regarding important interactions on different chromosomes. (A**) The Receiver Operating Characteristic curves of all enhancement methods regarding important interactions on chromosomes 18. **(B**) The Receiver Operating Characteristic curves of all enhancement methods regarding important interactions on chromosomes 19. **(C)** The Receiver Operating Characteristic curves of all enhancement methods regarding important interactions on chromosomes 20. **(D)** The Receiver Operating Characteristic curves of all enhancement methods regarding important interactions on chromosomes 21. **(E)** The Receiver Operating Characteristic curves of all enhancement methods regarding important interactions on chromosomes 22.


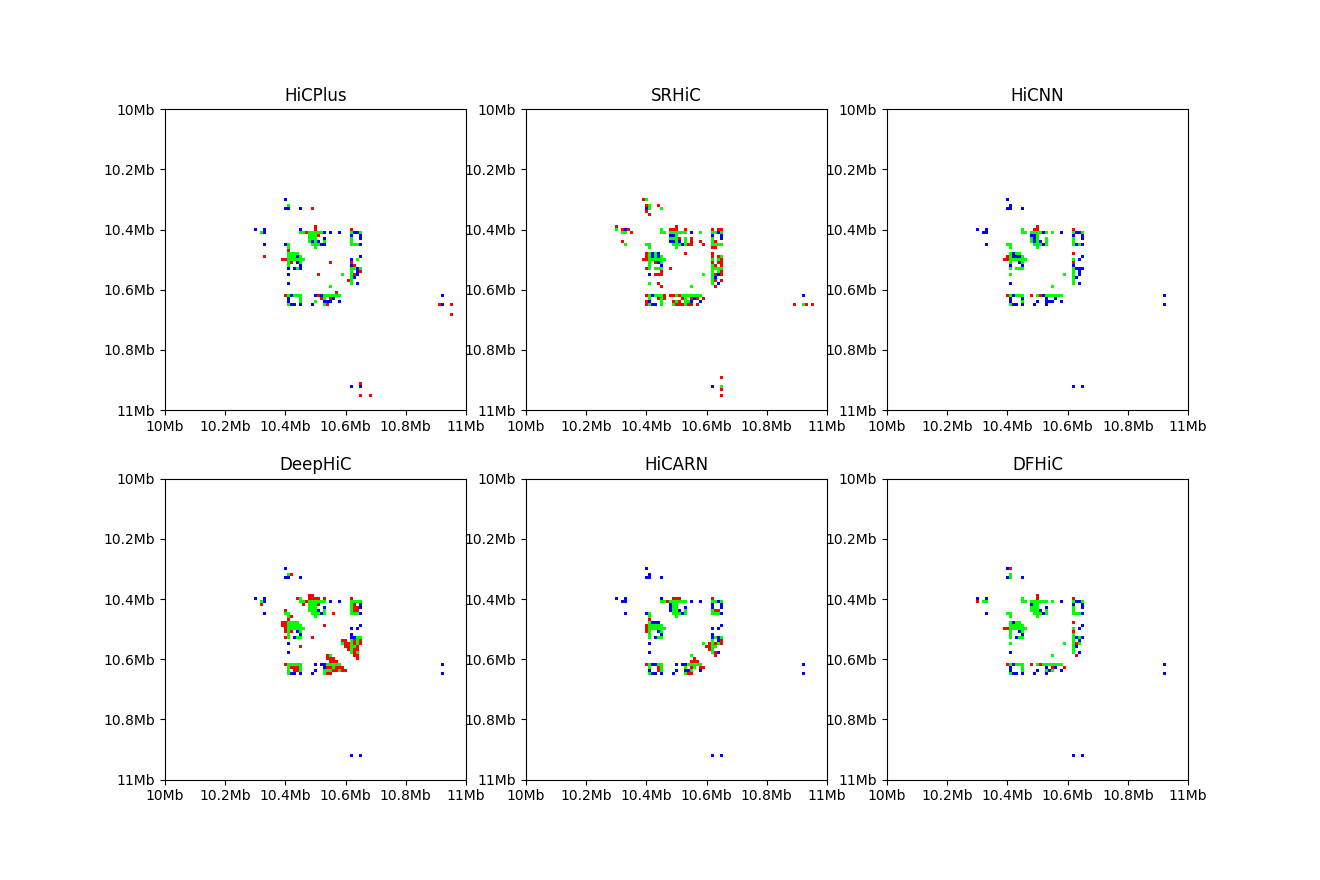


**Fig. S6.** **Visualization of significant interactions detected in chr20 (10-11Mb).** The blue part is the false negative significant interactions, the green part is the true positive significant interactions, and the red part is the false positive significant interactions.


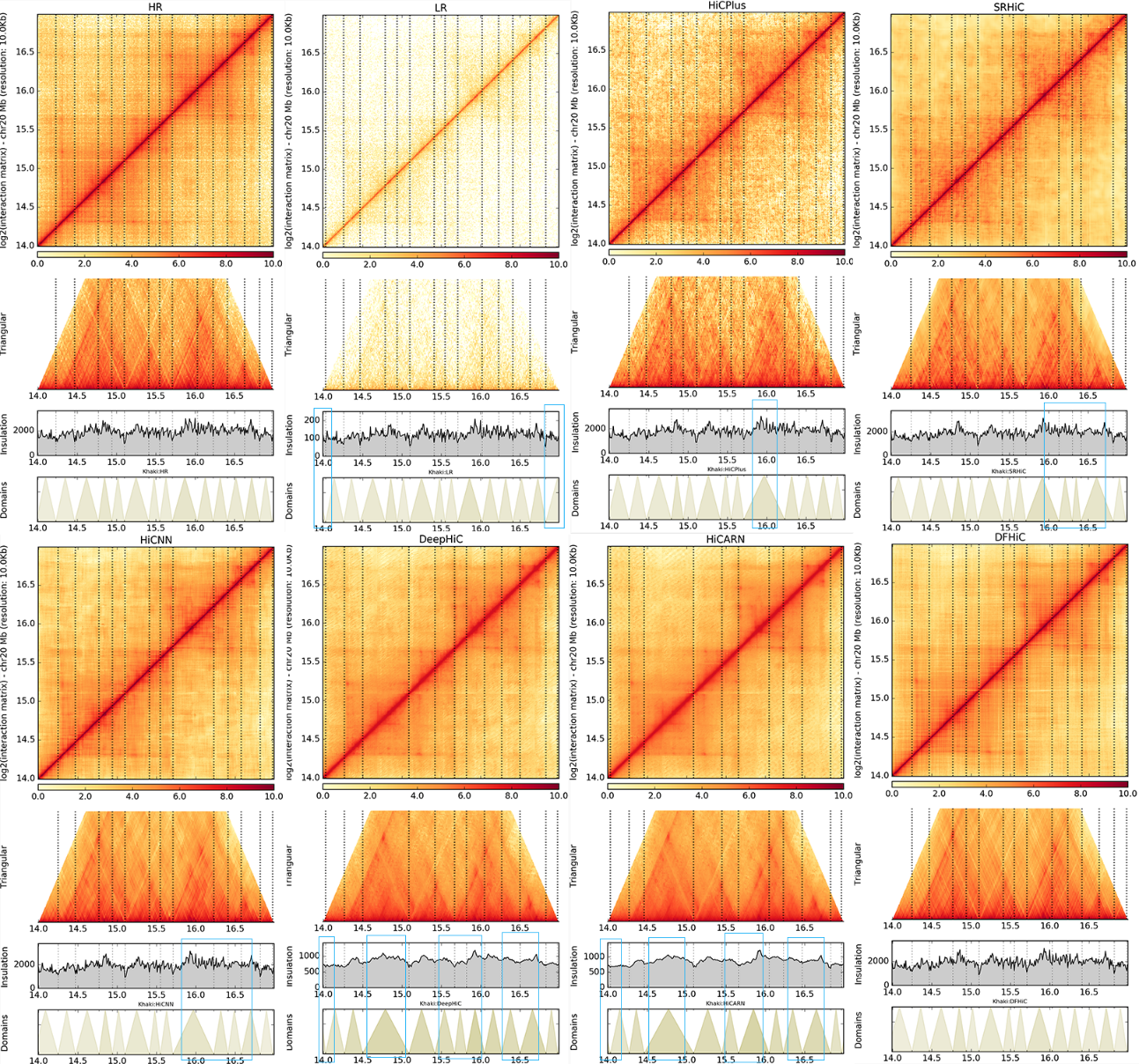


**Fig. S7.** **Visualization of Hi-C data and detected TAD on chr20(14Mb-17Mb) by HiCPlotter.** Fig. S7 shows more detailed TAD detection results and Insulation scores.


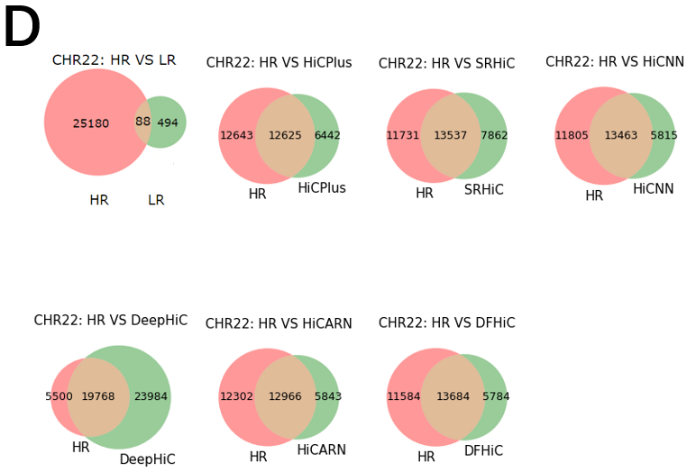

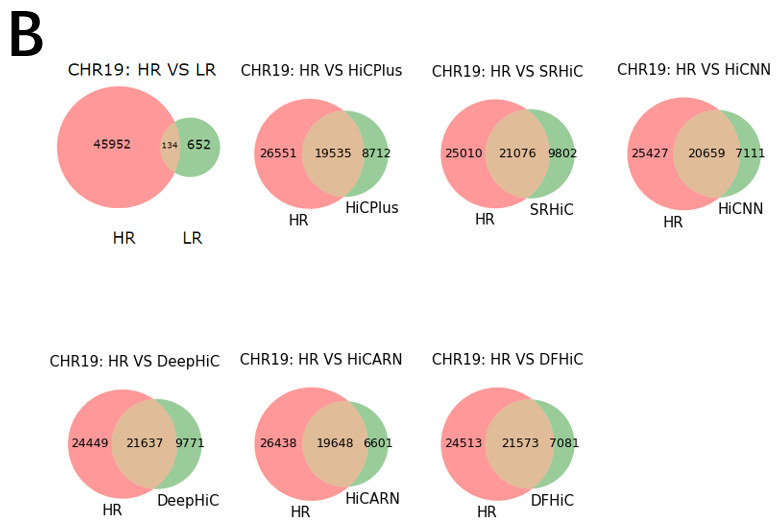

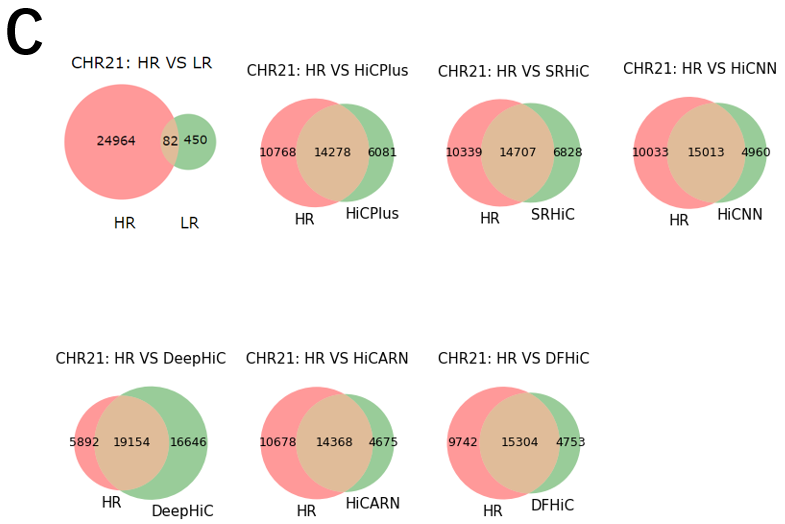

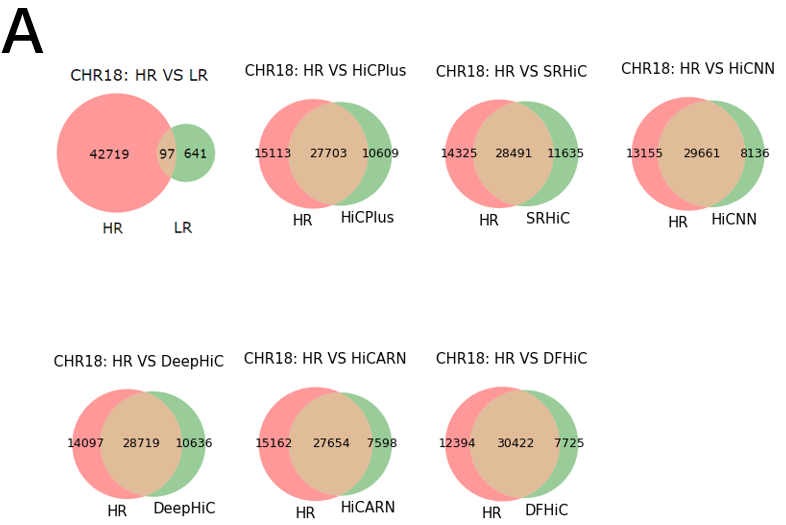


**Fig. S8. The number of significant interactions detected by Fit-Hi-C on different chromosomes recovered by different methods for actual low-resolution Hi-C data. (A)** Chromosome 18. **(B)** Chromosome 19. **(C)** Chromosome 21. **(D)** Chromosome 22.


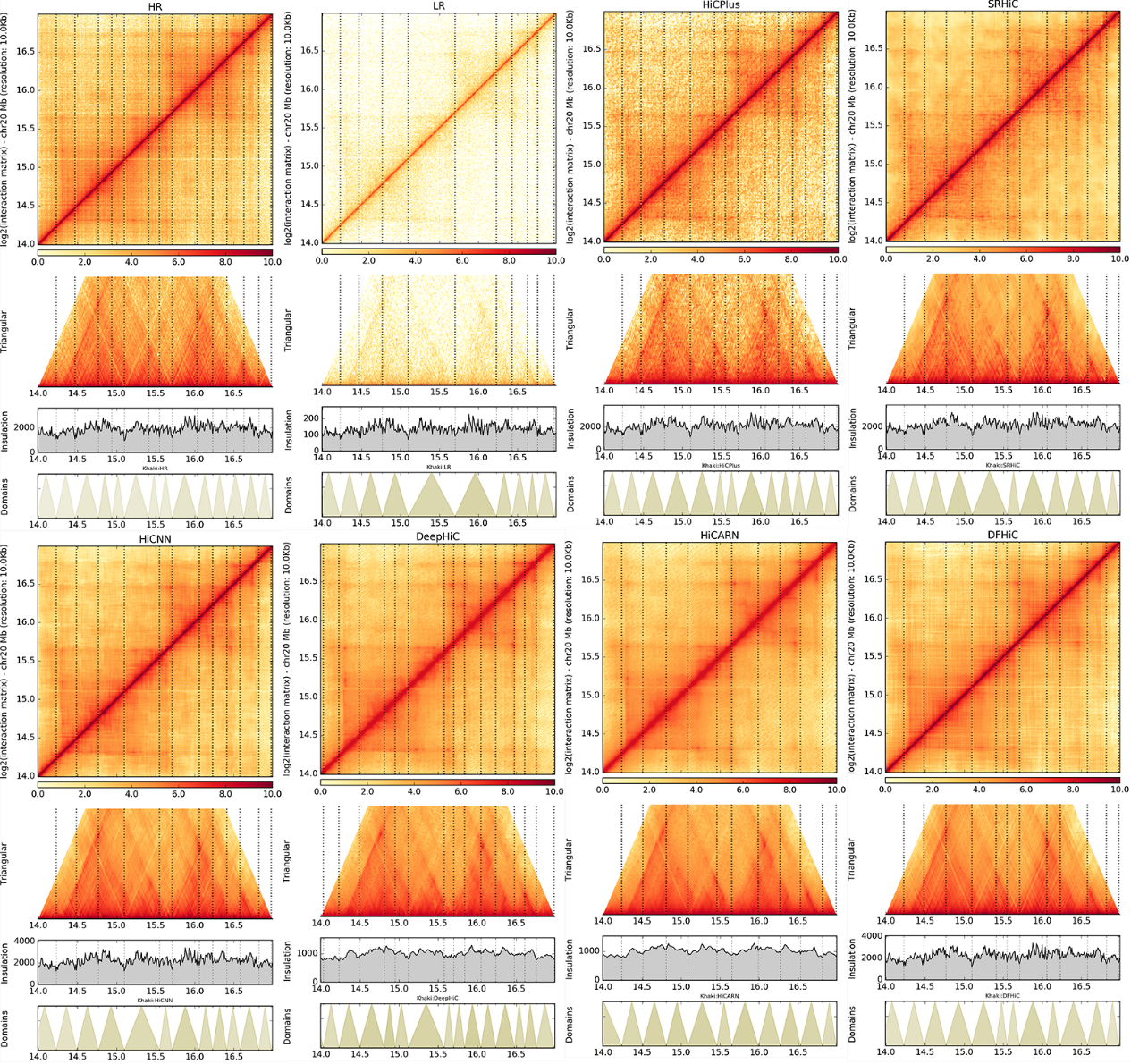


**Fig. S9.** **Visualization of Hi-C data for actual low-resolution and detected TAD on chr20(14Mb-17Mb) by HiCPlotter.**


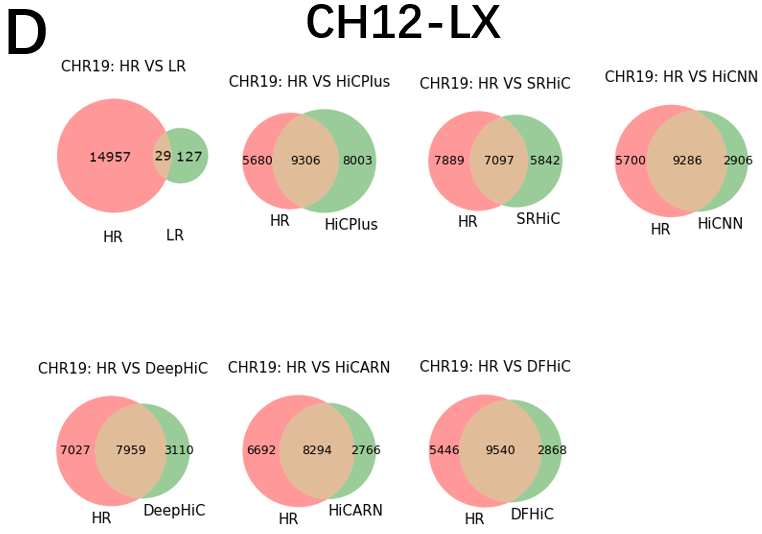

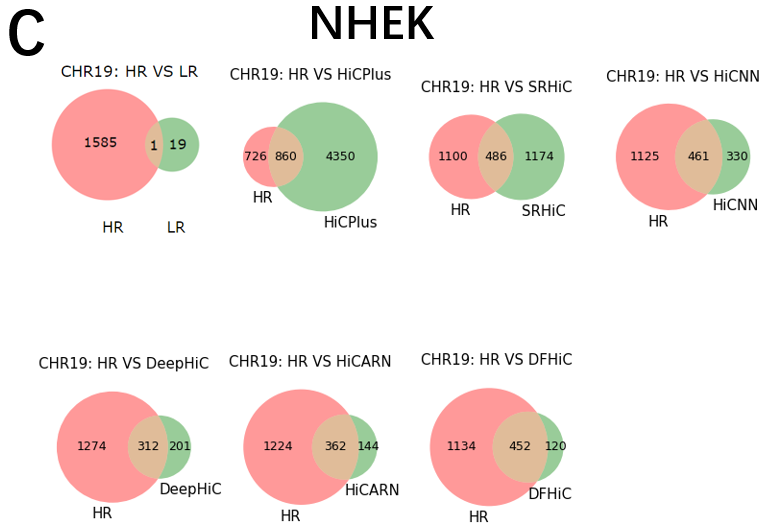

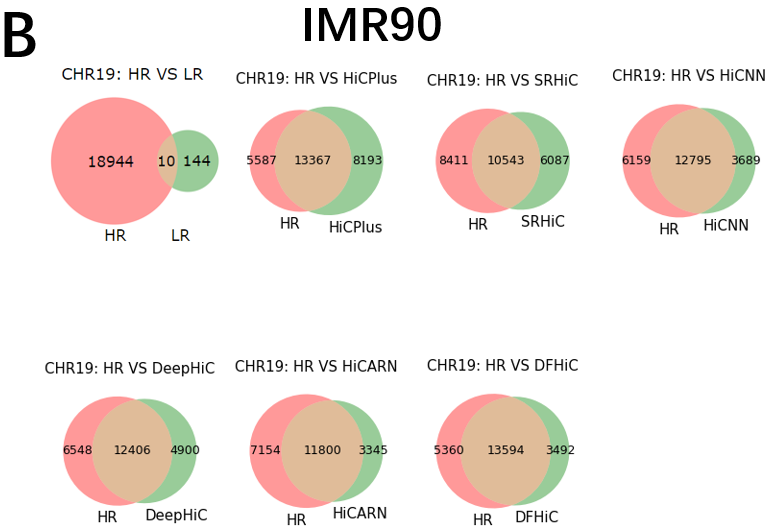

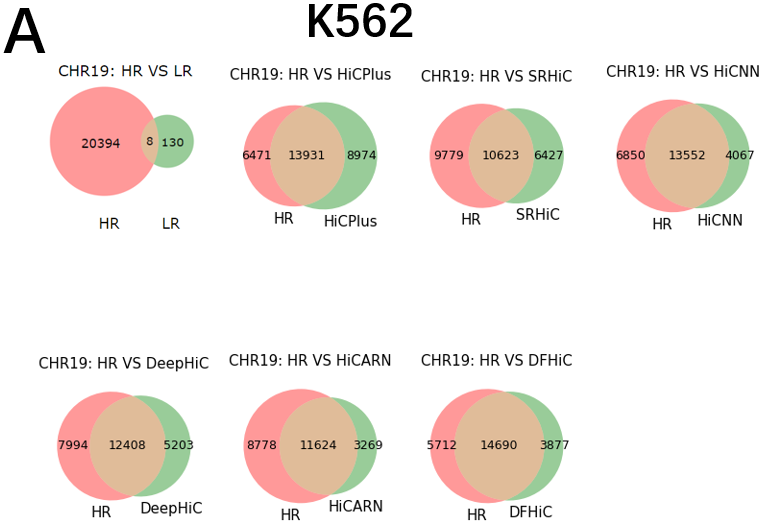


**Fig. S10. The number of significant interactions detected by Fit-Hi-C on chromosome 19 recovered by different methods for different cell types. (A)** Cell K562. **(B)** Cell IMR90. **(C)** Cell NHEK. **(D)** Cell CH12-LX.


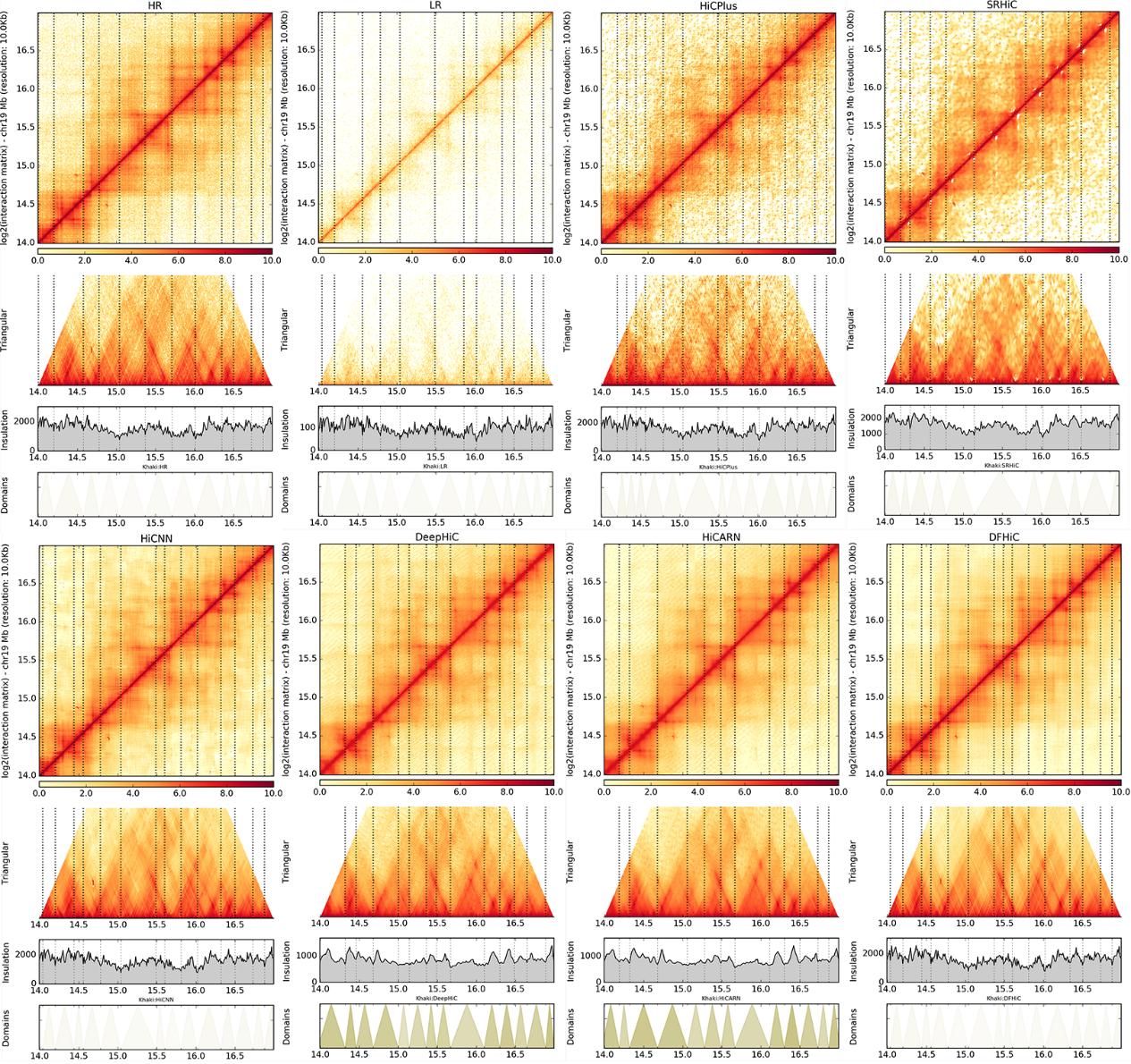


**Fig. S11.** **Visualization of Hi-C data for K562 and detected TAD on chr20(14Mb-17Mb) by HiCPlotter.**


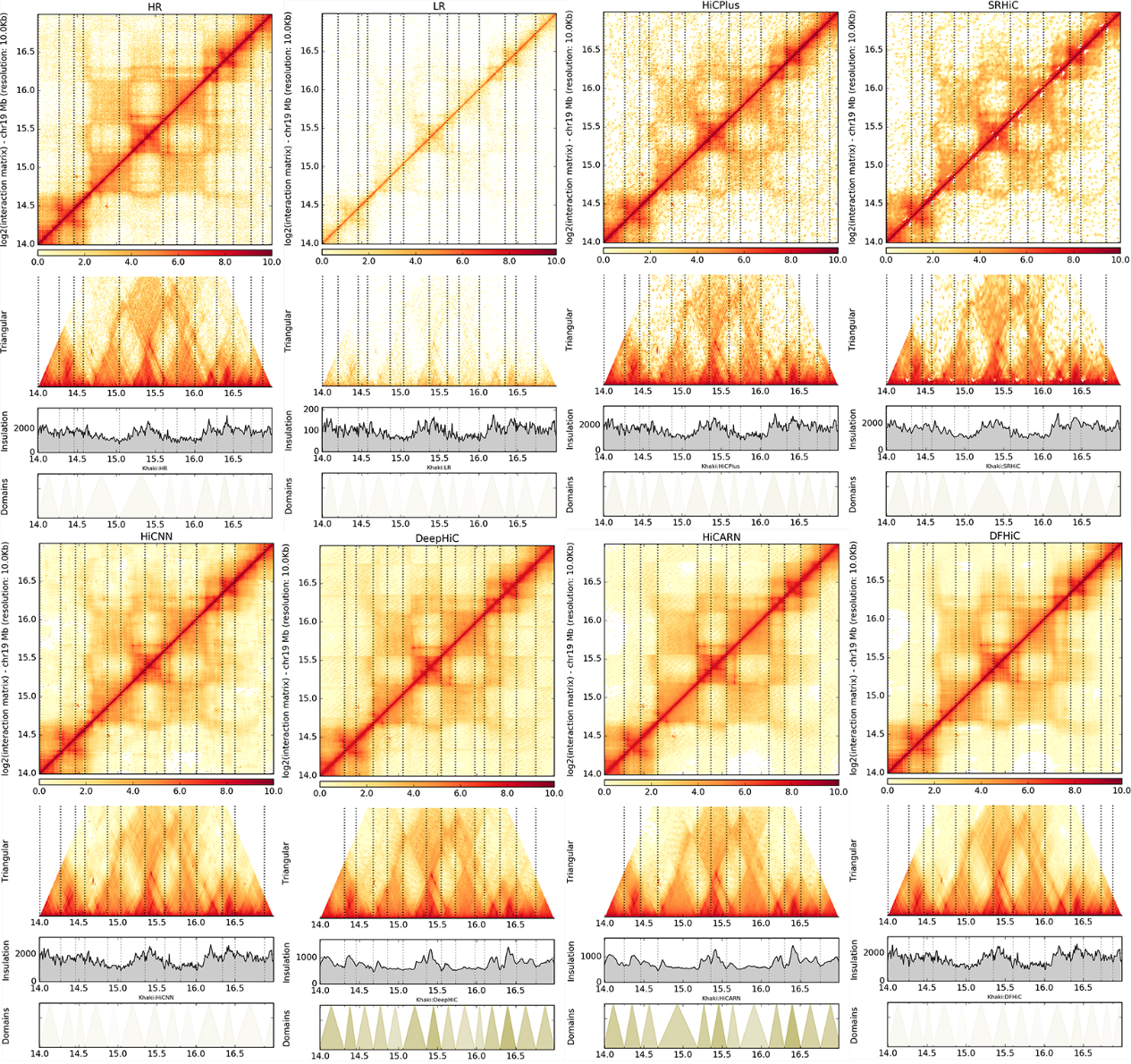


**Fig. S12.** **Visualization of Hi-C data for IMR90 and detected TAD on chr20(14Mb-17Mb) by HiCPlotter.**


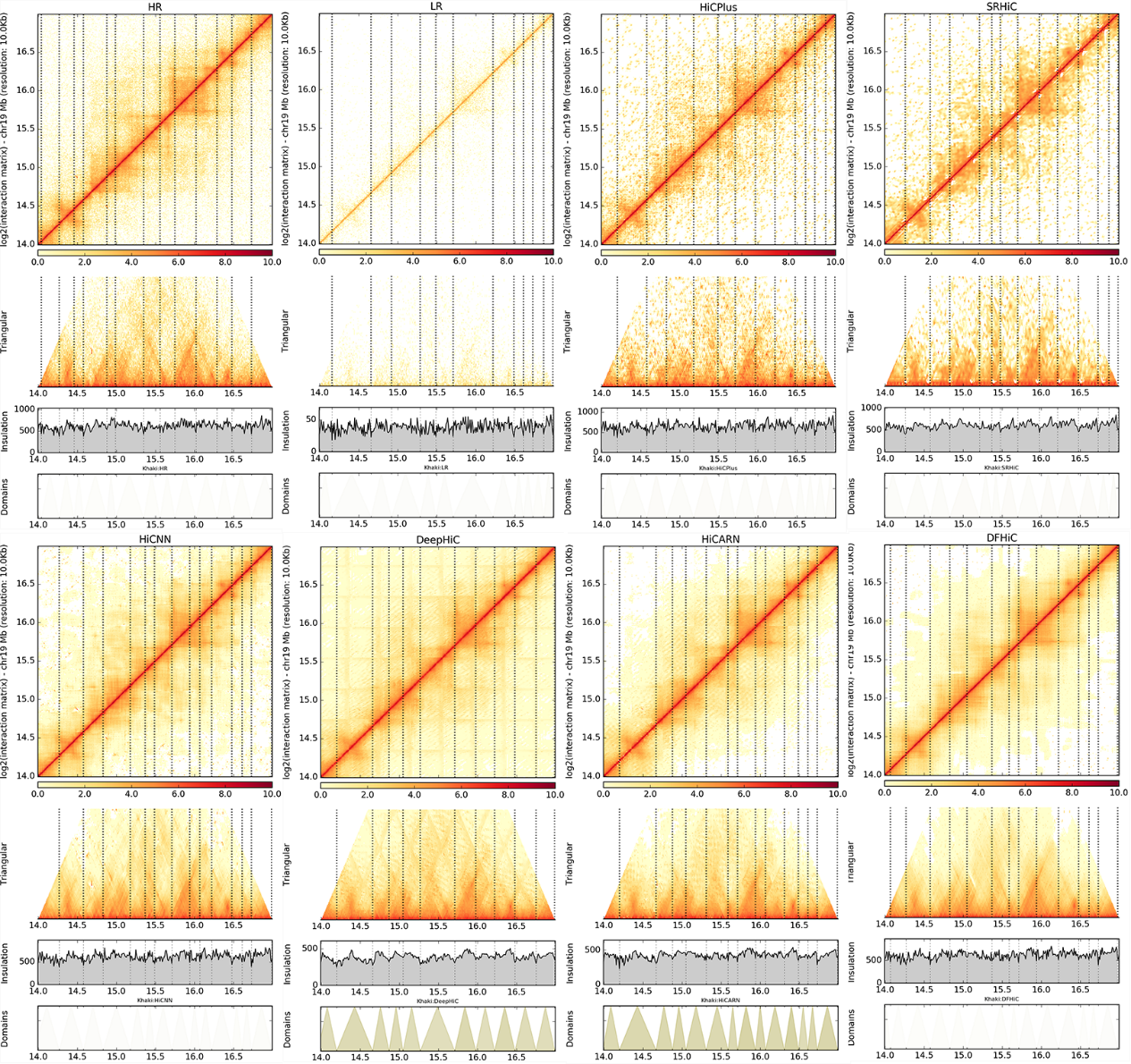


**Fig. S13.** **Visualization of Hi-C data for NHEK and detected TAD on chr20(14Mb-17Mb) by HiCPlotter.**


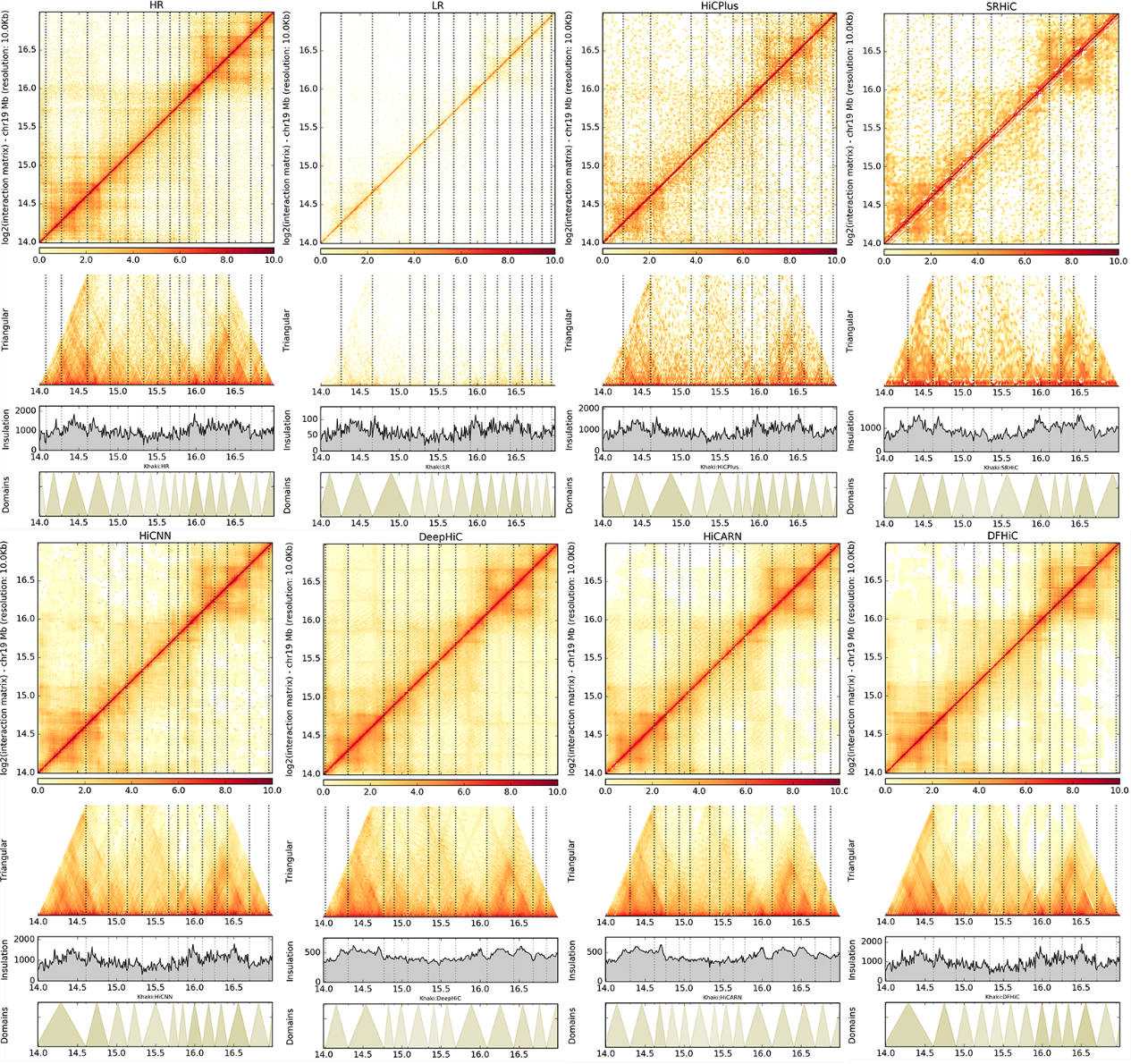


**Fig. S14.** **Visualization of Hi-C data for CH12-LX and detected TAD on chr20(14Mb-17Mb) by HiCPlotter.**


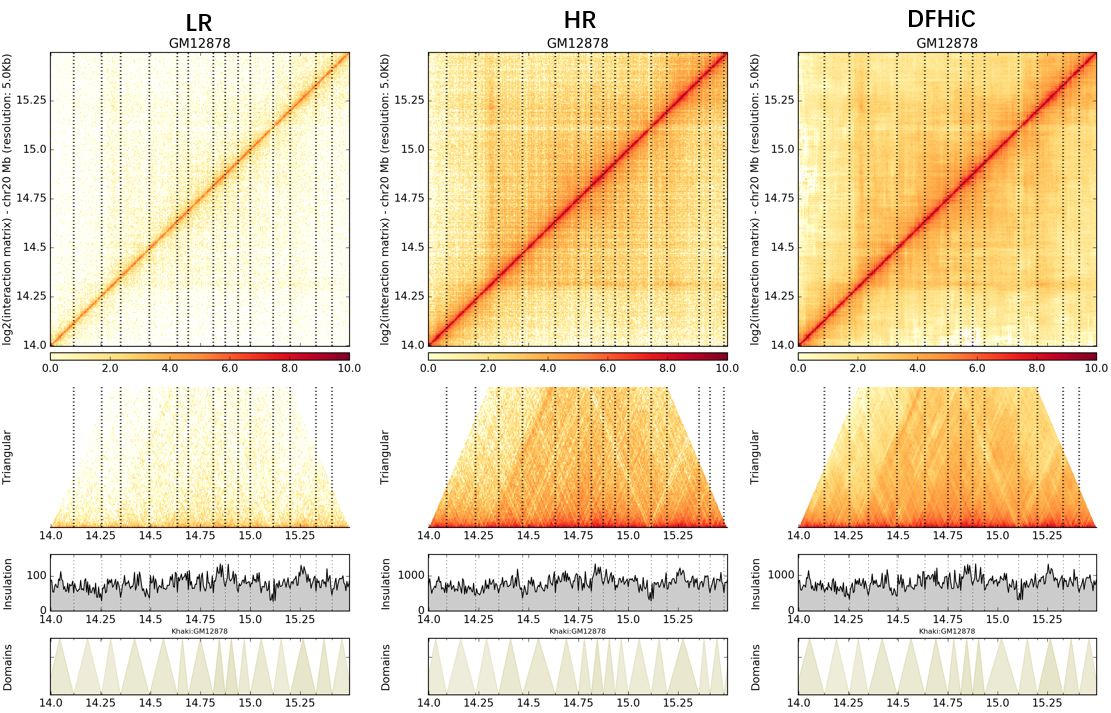


**Fig. S15.** **Visualization of Hi-C data(5kb) and detected TAD on chr20(14Mb-15.5Mb) by HiCPlotter.**

# Supplementary Tables

**Table S1. The details of all data sets in this study.**

| **Cell Line** | **Dataset** | **Chromosomes** |
| --- | --- | --- |
| GM12878(GSE63525) | Train | 1，2，3，4，5，6，7，8，9，10，11，12，13，14，15，16，17 |
|  | Test | 18，19，20，21，22 |
| K562(GSE63525) | Test | 18，19，20，21，22 |
| IMR90(GSE63525) | Test | 18，19，20，21，22 |
| NHEK(GSE63525) | Test | 18，19，20，21，22 |
| CH12-LX(GSE63525) | Test | 16，17，18，19 |

**Table S2. The comparison results of the all enhancement methods on the test set at different genomic distances.**

|  | **Model** | **Distance 0** | **Distance 40** | **Distance 80** | **Distance 120** |
| --- | --- | --- | --- | --- | --- |
| **MSE** | HiCPlus | 1212.55 | 137.93 | 25.31 | 9.81 |
|  | SRHiC | 2352.72 | 260.35 | 24.21 | 10.23 |
|  | HiCNN | 1260.65 | 181.86 | 18.21 | 8.27 |
|  | DeepHiC | 70156.83 | 181.74 | 18.56 | 8.88 |
|  | HiCARN | 70104.31 | 170.82 | 17.44 | 8.11 |
|  | **DFHiC** | **871.21** | **63.79** | **15.79** | **7.51** |
| **PSNR** | HiCPlus | 37.93 | 34.11 | 18.65 | 17.17 |
|  | SRHiC | 35.20 | 31.48 | 18.77 | 17.25 |
|  | HiCNN | 37.87 | 33.05 | 20.08 | 18.10 |
|  | DeepHiC | 20.78 | 32.56 | 19.97 | 17.83 |
|  | HiCARN | 20.79 | 32.77 | 20.25 | 18.23 |
|  | **DFHiC** | **39.39** | **37.18** | **20.71** | **18.56** |
| **SSIM** | HiCPlus | 0.82 | 0.48 | 0.29 | 0.20 |
|  | SRHiC | 0.79 | 0.45 | 0.23 | 0.11 |
|  | HiCNN | 0.84 | 0.50 | 0.30 | 0.23 |
|  | DeepHiC | 0.47 | 0.43 | 0.28 | 0.23 |
|  | HiCARN | 0.49 | 0.45 | 0.28 | 0.23 |
|  | **DFHiC** | **0.88** | **0.58** | **0.35** | **0.24** |

**Table S3. The comparison results of all method times and memory used. Time required for each epoch during training and memory required to run a Hi-C matrix of size 40x40.**

| **Methods** | **Per epoch** | **Total memory(40x40)** |
| --- | --- | --- |
| HiCPlus | 2.425s | 0.07MB |
| SRHiC | 16.478s | 2.74MB |
| HiCNN | 130.67s | 11.70MB |
| DeepHiC | 138.865s | 8.99MB |
| HiCARN | 168.34s | 19.93MB |
| DFHiC | 65.78s | 11.07MB |

**Table S4. The influence of dilated convolution and dilation rates on the method.**

| **Methods** | **MSE** | **PSNR** | **SSIM** |
| --- | --- | --- | --- |
| Vanilla_Conv | 16.568 | 20.56 | 0.219 |
| **DFHiC** | **14.201** | **21.133** | **0.342** |
| Dilation_Rate_5 | 15.436 | 20.936 | 0.334 |
| Dilation_Rate_7 | 16.816 | 20.606 | 0.324 |
| Dilation_Rate_9 | 17.026 | 20.521 | 0.321 |

**Table S5. The comparison results of dilated convolution and vanilla convolution. Time required for per epoch during training.**

| **Methods** | **Per epoch** |
| --- | --- |
| Vanilla_Conv | 40.725s |
| **DFHiC** | 65.786s |

**Table S6. The comparison results of training DFHiC with MSE and MAE as loss function, respectively.**

| **Loss functions** | **MSE** | **PSNR** | **SSIM** |
| --- | --- | --- | --- |
| **MAE Loss** | **14.201** | **21.133** | **0.342** |
| MSE Loss | 15.673 | 20.782 | 0.302 |

**Table S7.** **The comparison results of F1 scores recovered by all methods for significant interactions of chromosomes.**

| **Model** | **Chr18** | **Chr19** | **Chr20** | **Chr21** | **Chr22** |
| --- | --- | --- | --- | --- | --- |
| LR | 0.004 | 0.015 | 0.013 | 0.011 | 0.012 |
| HiCPlus | 0.759 | 0.783 | 0.801 | 0.808 | 0.788 |
| SRHiC | 0.747 | 0.764 | 0.781 | 0.792 | 0.765 |
| HiCNN | 0.809 | 0.829 | 0.846 | 0.846 | 0.835 |
| DeepHiC | 0.759 | 0.747 | 0.784 | 0.73 | 0.598 |
| HiCARN | 0.777 | 0.765 | 0.816 | 0.82 | 0.778 |
| **DFHiC** | **0.83** | **0.849** | **0.867** | **0.869** | **0.86** |

**Table S8.** **The comparison results of false positive rate recovered by all methods for significant interactions on chromosomes 18-22.**

| **Model** | **Chr18** | **Chr19** | **Chr20** | **Chr21** | **Chr22** | **Avg** |
| --- | --- | --- | --- | --- | --- | --- |
| LR | 91% | 76.5% | 82.1% | 87.3% | 84.5% | 84.3% |
| HiCPlus | 23.2% | 19.7% | 19.4% | 19.4% | 20.2% | 20.4% |
| SRHiC | 26.9% | 25.2% | 23.9% | 22.4% | 26.5% | 25% |
| HiCNN | 17.7% | 16.2% | 15.1% | 15.8% | 16.2% | 16.2% |
| DeepHiC | 24.7% | 29.1% | 25.3% | 41% | 56.7% | 35.4% |
| HiCARN | 18.9% | 33.3% | 26.3% | 44.6% | 61.3% | 36.9% |
| **DFHiC** | **16.5%** | **14%** | **12.7%** | **13.2%** | **13.3%** | **13.9%** |

**Table S9. The mean value of the p-values corresponding to the false positive significant interactions generated by all enhancement methods on chromosomes 18-22.**

| **Methods** | **chr18** | **chr19** | **chr20** | **chr21** | **chr22** |
| --- | --- | --- | --- | --- | --- |
| HiCPlus | 2.45E-08 | 3.32E-08 | 2.73E-08 | 2.88E-08 | 2.88E-08 |
| HiCNN | 2.94E-08 | 3.79E-08 | 3.26E-08 | 3.40E-08 | 3.30E-08 |
| SRHiC | 2.21E-08 | 2.69E-08 | 2.69E-08 | 2.80E-08 | 2.68E-08 |
| DeepHiC | 2.42E-08 | 3.22E-08 | 2.43E-08 | 2.55E-08 | 2.48E-08 |
| HiCARN | 2.48E-08 | 3.52E-08 | 2.84E-08 | 2.98E-08 | 3.11E-08 |
| DFHiC | 3.23E-08 | 4.55E-08 | 3.98E-08 | 4.00E-08 | 3.94E-08 |

**Table S10. The mean value of the p-values corresponding to the false negative significant interactions generated by all enhancement methods on chromosomes 18-22.**

| **Methods** | **chr18** | **chr19** | **chr20** | **chr21** | **chr22** |
| --- | --- | --- | --- | --- | --- |
| HiCPlus | 2.48E-08 | 3.42E-08 | 2.46E-08 | 3.04E-08 | 2.74E-08 |
| HiCNN | 2.95E-08 | 4.13E-08 | 3.05E-08 | 3.86E-08 | 3.38E-08 |
| SRHiC | 2.44E-08 | 3.30E-08 | 2.41E-08 | 2.89E-08 | 2.57E-08 |
| DeepHiC | 2.50E-08 | 3.22E-08 | 2.62E-08 | 4.03E-08 | 3.55E-08 |
| HiCARN | 2.58E-08 | 3.10E-08 | 2.78E-08 | 3.36E-08 | 2.48E-08 |
| DFHiC | 3.20E-08 | 4.52E-08 | 3.39E-08 | 4.12E-08 | 3.78E-08 |

**Table S11. The mean value of the p-values corresponding to the true positive significant interactions generated by all enhancement methods on chromosomes 18-22.**

| **Methods** | **chr18** | **chr19** | **chr20** | **chr21** | **chr22** |
| --- | --- | --- | --- | --- | --- |
| HiCPlus | 5.91E-09 | 7.90E-09 | 5.47E-09 | 5.68E-09 | 6.02E-09 |
| HiCNN | 6.12E-09 | 7.17E-09 | 5.82E-09 | 5.78E-09 | 5.82E-09 |
| SRHiC | 5.80E-09 | 6.85E-09 | 5.84E-09 | 5.87E-09 | 6.23E-09 |
| DeepHiC | 6.27E-09 | 8.15E-09 | 5.26E-09 | 2.77E-09 | 1.89E-09 |
| HiCARN | 6.23E-09 | 1.02E-08 | 5.70E-09 | 5.84E-09 | 8.57E-09 |
| DFHiC | 6.26E-09 | 7.76E-09 | 5.75E-09 | 6.05E-09 | 5.84E-09 |

**Table S12. The HiCRep and HiC-Spetor’s scores of the biological reproducibility comparison of all enhanced Hi-C matrices and high-resolution Hi-C matrices on chromosomes 18-22 in GM12878.**

|  | **Model** | **Chr18** | **Chr19** | **Chr20** | **Chr21** | **Chr22** | **Avg** |
| --- | --- | --- | --- | --- | --- | --- | --- |
| **HiCRep** | HiCPlus | 0.924 | 0.963 | 0.937 | 0.933 | 0.977 | 0.947 |
|  | SRHiC | 0.91 | 0.962 | 0.937 | 0.941 | 0.977 | 0.945 |
|  | HiCNN | **0.947** | 0.974 | 0.956 | 0.952 | 0.983 | 0.962 |
|  | DeepHiC | 0.94 | 0.955 | 0.954 | 0.956 | 0.973 | 0.956 |
|  | **HiCARN** | 0.953 | 0.966 | **0.963** | **0.964** | 0.977 | **0.965** |
|  | DFHiC | 0.942 | **0.975** | 0.957 | 0.948 | **0.985** | 0.961 |
| **HiC-Spetor** | **HiCPlus** | 0.548 | 0.388 | **0.455** | 0.263 | **0.72** | **0.475** |
|  | SRHiC | 0.251 | 0.256 | 0.272 | 0.194 | 0.422 | 0.279 |
|  | HiCNN | 0.383 | 0.344 | 0.335 | 0.24 | 0.635 | 0.387 |
|  | DeepHiC | 0.375 | 0.31 | 0.37 | 0.215 | 0.379 | 0.33 |
|  | HiCARN | 0.267 | 0.327 | 0.338 | **0.283** | 0.367 | 0.316 |
|  | DFHiC | **0.609** | **0.788** | 0.419 | 0.256 | 0.277 | 0.47 |

**Table S13.** **The comparison results of F1 scores recovered by all methods for significant interactions of chromosomes for actual low resolution Hi-C data.**

| **Model** | **Chr18** | **Chr19** | **Chr20** | **Chr21** | **Chr22** |
| --- | --- | --- | --- | --- | --- |
| LR | 0.004 | 0.006 | 0.009 | 0.006 | 0.007 |
| HiCPlus | 0.683 | 0.526 | 0.659 | 0.629 | 0.57 |
| SRHiC | 0.687 | 0.548 | 0.662 | 0.632 | 0.58 |
| HiCNN | 0.736 | 0.559 | 0.7 | 0.67 | 0.605 |
| DeepHiC | 0.699 | 0.558 | 0.682 | 0.63 | 0.573 |
| HiCARN | 0.708 | 0.543 | 0.689 | 0.652 | 0.588 |
| **DFHiC** | **0.752** | **0.577** | **0.712** | **0.679** | **0.612** |

**Table S14.** **The comparison results of false positive rate recovered by all methods for significant interactions of chromosomes for actual low resolution Hi-C data.**

| **Model** | **Chr18** | **Chr19** | **Chr20** | **Chr21** | **Chr22** | **Avg** |
| --- | --- | --- | --- | --- | --- | --- |
| LR | 86.9% | 83% | 81.8% | 84.6% | 84.9% | 84.2% |
| HiCPlus | 27.7% | 30.8% | 24% | 29.9% | 33.8% | 29.2% |
| SRHiC | 29% | 31.7% | 26.8% | 31.7% | 36.7% | 31.2% |
| HiCNN | 21.5% | 25.6% | 18.9% | 24.8% | 30.2% | 24.2% |
| DeepHiC | 27% | 31.1% | 24.3% | 46.5% | 54.8% | 36.7% |
| HiCARN | 21.6% | 25.1% | 18.9% | 24.5% | 31.1% | 24.2% |
| **DFHiC** | **20.3%** | **24.7%** | **17.5%** | **23.7%** | **29.7%** | **23.2%** |

**Table S15. The comparison results of TAD boundary detected by the enhancement methods on chromosomes 18 - 22 in GM12878 for actual low-resolution Hi-C data.**

| **Methods** | **Chr18** | **Chr19** | **Chr20** | **Chr21** | **Chr22** | **Avg** |
| --- | --- | --- | --- | --- | --- | --- |
| LR | 0.058 | 0.067 | 0.052 | 0.058 | 0.093 | 0.065 |
| HiCPlus | 0.377 | 0.4 | 0.41 | 0.41 | 0.429 | 0.405 |
| SRHiC | 0.46 | 0.444 | 0.41 | 0.482 | 0.527 | 0.465 |
| HiCNN | 0.537 | 0.514 | 0.532 | 0.554 | **0.566** | 0.541 |
| DeepHiC | 0.47 | 0.457 | 0.495 | 0.59 | 0.532 | 0.509 |
| HiCARN | 0.498 | 0.517 | 0.505 | **0.597** | 0.498 | 0.523 |
| **DFHiC** | **0.559** | **0.559** | **0.541** | 0.583 | 0.551 | **0.559** |

**Table S16. The Reproducibility Scores of all enhanced Hi-C matrices for actual low-resolution Hi-C data and high-resolution Hi-C matrices on chromosomes 18-22 in GM12878.**

|  | **Model** | **Chr18** | **Chr19** | **Chr20** | **Chr21** | **Chr22** | **Avg** |
| --- | --- | --- | --- | --- | --- | --- | --- |
| **GenomeDISCO** | **HiCPlus** | **0.874** | **0.859** | **0.869** | **0.862** | **0.858** | **0.864** |
|  | SRHiC | 0.478 | 0.609 | 0.593 | 0.499 | 0.420 | 0.520 |
|  | HiCNN | 0.779 | 0.817 | 0.801 | 0.811 | 0.833 | 0.808 |
|  | DeepHiC | 0.449 | 0.379 | 0.404 | 0.127 | 0.068 | 0.285 |
|  | HiCARN | 0.738 | 0.788 | 0.794 | 0.798 | 0.793 | 0.782 |
|  | DFHiC | 0.807 | 0.766 | 0.786 | 0.805 | 0.762 | 0.785 |
| **QuASAR-Rep** | HiCPlus | 0.898 | 0.904 | 0.917 | 0.903 | 0.911 | 0.907 |
|  | SRHiC | 0.911 | 0.935 | 0.940 | 0.907 | 0.936 | 0.926 |
|  | HiCNN | 0.925 | 0.938 | **0.945** | 0.921 | 0.945 | **0.935** |
|  | **DeepHiC** | **0.926** | 0.931 | 0.938 | **0.926** | 0.939 | 0.932 |
|  | HiCARN | 0.924 | 0.934 | 0.938 | 0.924 | 0.941 | 0.932 |
|  | **DFHiC** | 0.920 | **0.939** | **0.945** | 0.922 | **0.948** | **0.935** |
| **HiCRep** | HiCPlus | 0.878 | 0.837 | 0.836 | 0.824 | 0.908 | 0.857 |
|  | SRHiC | 0.865 | **0.855** | 0.848 | 0.863 | 0.918 | 0.870 |
|  | HiCNN | 0.901 | 0.850 | 0.849 | 0.837 | 0.913 | 0.870 |
|  | **DeepHiC** | 0.900 | 0.830 | **0.863** | **0.887** | **0.926** | **0.881** |
|  | HiCARN | **0.908** | 0.846 | 0.860 | 0.865 | 0.921 | 0.880 |
|  | DFHiC | 0.896 | 0.838 | 0.841 | 0.830 | 0.906 | 0.862 |
| **HiC-Spetor** | HiCPlus | 0.392 | 0.257 | 0.356 | 0.420 | 0.464 | 0.378 |
|  | SRHiC | 0.302 | **0.338** | 0.362 | 0.301 | 0.343 | 0.329 |
|  | HiCNN | 0.242 | 0.281 | 0.283 | 0.354 | 0.397 | 0.311 |
|  | DeepHiC | 0.262 | 0.228 | 0.244 | 0.132 | 0.318 | 0.237 |
|  | HiCARN | 0.265 | 0.303 | 0.414 | 0.450 | 0.423 | 0.371 |
|  | **DFHiC** | **0.492** | 0.287 | **0.609** | **0.743** | **0.674** | **0.561** |

**Table S17. The comparison results of the enhancement methods on the test set for different cell types and different species.**

|  | **Model** | **MSE** | **PSNR** | **SSIM** |
| --- | --- | --- | --- | --- |
| **NHEK** | HiCPlus | 3.862 | 12.315 | 0.17 |
|  | SRHiC | 1.965 | 15.574 | 0.065 |
|  | HiCNN | 1.642 | 15.82 | 0.153 |
|  | DeepHiC | 1.665 | 15.553 | 0.134 |
|  | HiCARN | 1.252 | 17.188 | 0.168 |
|  | **DFHiC** | **1.23** | **16.83** | **0.175** |
| **CH12-LX** | HiCPlus | 2.403 | 13.022 | 0.137 |
|  | SRHiC | 1.362 | 16.021 | 0.029 |
|  | HiCNN | 1.413 | 15 | 0.136 |
|  | DeepHiC | 1.201 | 15.228 | 0.105 |
|  | **HiCARN** | **0.794** | **17.725** | **0.159** |
|  | DFHiC | 0.95 | 16.57 | 0.143 |

**Table S18.** **The comparison results of false positive rate recovered by all methods for significant interactions of chromosome 19 on different cell types.**

| **Model** | **K562** | **IMR90** | **NHEK** | **CH12-LX** | **Avg** |
| --- | --- | --- | --- | --- | --- |
| LR | 94.2% | 93.5% | 95% | 81.4% | 91% |
| HiCPlus | 39.2% | 38% | 83.5% | 46.2% | 51.7% |
| SRHiC | 37.7% | 36.6% | 70.7% | 45.2% | 47.5% |
| HiCNN | 23.1% | 22.4% | 41.7% | 23.8% | 27.7% |
| DeepHiC | 29.5% | 28.3% | 39.2% | 28.1% | 31.3% |
| HiCARN | 21.9% | 22.1% | 28.6% | 25% | 24.4% |
| **DFHiC** | **20.9%** | **20.4%** | **21%** | **23.1%** | **21.3%** |

**Table S19. The comparison results of TAD boundary detected by the enhancement methods on chromosomes 18 - 22 in GM12878 for K562 cell.**

| **Methods** | **Chr18** | **Chr19** | **Chr20** | **Chr21** | **Chr22** | **Avg** |
| --- | --- | --- | --- | --- | --- | --- |
| HiCPlus | 0.274 | 0.427 | 0.393 | 0.413 | 0.435 | 0.388 |
| SRHiC | 0.345 | 0.325 | 0.315 | 0.355 | 0.386 | 0.345 |
| HiCNN | 0.469 | 0.529 | 0.496 | 0.507 | 0.57 | 0.514 |
| DeepHiC | 0.438 | 0.454 | 0.507 | 0.471 | 0.516 | 0.477 |
| HiCARN | 0.478 | 0.515 | 0.493 | **0.558** | 0.576 | 0.524 |
| **DFHiC** | **0.5** | **0.556** | **0.537** | 0.486 | **0.582** | **0.532** |

**Table S20. The comparison results of TAD boundary detected by the enhancement methods on chromosomes 18 - 22 in GM12878 for IMR90 cell.**

| **Methods** | **Chr18** | **Chr19** | **Chr20** | **Chr21** | **Chr22** | **Avg** |
| --- | --- | --- | --- | --- | --- | --- |
| HiCPlus | 0.368 | 0.381 | 0.37 | 0.415 | 0.399 | 0.387 |
| SRHiC | 0.299 | 0.292 | 0.322 | 0.327 | 0.293 | 0.307 |
| HiCNN | 0.475 | 0.46 | 0.548 | 0.558 | 0.576 | 0.523 |
| DeepHiC | 0.465 | **0.524** | 0.557 | 0.531 | 0.535 | 0.522 |
| HiCARN | 0.5 | 0.517 | 0.551 | 0.592 | 0.57 | 0.546 |
| **DFHiC** | **0.522** | **0.524** | **0.63** | **0.6** | **0.616** | **0.576** |

**Table S21. The comparison results of TAD boundary detected by the enhancement methods on chromosomes 18 - 22 in GM12878 for NHEK cell.**

| **Methods** | **Chr18** | **Chr19** | **Chr20** | **Chr21** | **Chr22** | **Avg** |
| --- | --- | --- | --- | --- | --- | --- |
| HiCPlus | 0.11 | 0.127 | 0.116 | 0.099 | 0.164 | 0.123 |
| SRHiC | 0.082 | 0.068 | 0.079 | 0.092 | 0.03 | 0.07 |
| **HiCNN** | 0.392 | 0.326 | 0.414 | **0.373** | **0.448** | **0.391** |
| DeepHiC | 0.399 | **0.339** | 0.418 | 0.345 | 0.412 | 0.383 |
| HiCARN | 0.361 | 0.326 | 0.401 | 0.31 | 0.382 | 0.356 |
| DFHiC | **0.423** | **0.339** | **0.438** | 0.359 | 0.37 | 0.386 |

**Table S22. The comparison results of TAD boundary detected by the enhancement methods on chromosomes 16 - 19 in GM12878 for CH12-LX cell.**

| **Methods** | **Chr16** | **Chr17** | **Chr18** | **Chr19** | **Avg** |
| --- | --- | --- | --- | --- | --- |
| HiCPlus | 0.116 | 0.114 | 0.07 | 0.147 | 0.112 |
| SRHiC | 0.058 | 0.068 | 0.07 | 0.074 | 0.068 |
| HiCNN | 0.447 | 0.5 | 0.394 | 0.5 | 0.46 |
| DeepHiC | 0.444 | 0.394 | 0.423 | 0.426 | 0.422 |
| HiCARN | 0.495 | 0.477 | 0.385 | 0.426 | 0.446 |
| **DFHiC** | **0.502** | **0.519** | **0.39** | **0.547** | **0.489** |

**Table S23. The Reproducibility Scores of all enhanced Hi-C matrices and high-resolution Hi-C matrices on chromosomes 18-22 in K562.**

|  | **Model** | **Chr18** | **Chr19** | **Chr20** | **Chr21** | **Chr22** | **Avg** |
| --- | --- | --- | --- | --- | --- | --- | --- |
| **GenomeDISCO** | **HiCPlus** | **0.860** | **0.870** | **0.870** | **0.880** | **0.880** | **0.872** |
|  | SRHiC | 0.850 | 0.860 | 0.858 | 0.858 | 0.863 | 0.858 |
|  | HiCNN | 0.750 | 0.718 | 0.727 | 0.742 | 0.735 | 0.734 |
|  | DeepHiC | 0.112 | 0.036 | 0.057 | -0.060 | -0.197 | -0.010 |
|  | HiCARN | 0.416 | 0.627 | 0.550 | 0.618 | 0.611 | 0.564 |
|  | DFHiC | 0.753 | 0.927 | 0.775 | 0.818 | 0.807 | 0.816 |
| **QuASAR-Rep** | HiCPlus | 0.785 | 0.860 | 0.834 | 0.850 | 0.896 | 0.845 |
|  | SRHiC | 0.806 | 0.870 | 0.856 | 0.871 | 0.907 | 0.862 |
|  | HiCNN | 0.870 | 0.929 | 0.907 | 0.909 | **0.931** | 0.909 |
|  | DeepHiC | 0.881 | 0.927 | 0.917 | 0.924 | 0.880 | 0.906 |
|  | **HiCARN** | **0.890** | **0.933** | **0.922** | **0.925** | 0.902 | **0.914** |
|  | DFHiC | 0.869 | 0.936 | 0.912 | 0.910 | 0.928 | 0.911 |
| **HiCRep** | HiCPlus | 0.906 | 0.896 | 0.889 | 0.895 | **0.992** | 0.916 |
|  | SRHiC | 0.893 | 0.881 | 0.880 | 0.884 | 0.981 | 0.904 |
|  | **HiCNN** | 0.913 | 0.906 | 0.902 | 0.913 | 0.991 | **0.925** |
|  | DeepHiC | 0.897 | 0.888 | 0.882 | 0.900 | 0.976 | 0.909 |
|  | HiCARN | **0.931** | **0.912** | **0.921** | **0.936** | 0.985 | 0.937 |
|  | DFHiC | 0.913 | 0.894 | 0.890 | 0.900 | 0.991 | 0.918 |
| **HiC-Spetor** | **HiCPlus** | 0.551 | 0.450 | **0.857** | **0.705** | 0.576 | **0.628** |
|  | SRHiC | **0.709** | 0.394 | 0.550 | 0.578 | **0.623** | 0.571 |
|  | HiCNN | 0.411 | **0.508** | 0.626 | 0.362 | 0.327 | 0.447 |
|  | DeepHiC | 0.163 | 0.308 | 0.135 | 0.126 | 0.150 | 0.176 |
|  | HiCARN | 0.179 | 0.383 | 0.273 | 0.139 | 0.333 | 0.261 |
|  | DFHiC | 0.325 | 0.358 | 0.147 | 0.232 | 0.362 | 0.285 |

**Table S24. The Reproducibility Scores of all enhanced Hi-C matrices and high-resolution Hi-C matrices on chromosomes 18-22 in IMR90.**

|  | **Model** | **Chr18** | **Chr19** | **Chr20** | **Chr21** | **Chr22** | **Avg** |
| --- | --- | --- | --- | --- | --- | --- | --- |
| **GenomeDISCO** | **HiCPlus** | **0.874** | **0.852** | **0.869** | **0.872** | **0.867** | **0.867** |
|  | SRHiC | 0.865 | 0.840 | 0.858 | 0.852 | 0.851 | 0.853 |
|  | HiCNN | 0.743 | 0.710 | 0.727 | 0.743 | 0.720 | 0.729 |
|  | DeepHiC | 0.230 | 0.081 | 0.153 | -0.061 | -0.162 | 0.048 |
|  | HiCARN | 0.480 | 0.593 | 0.624 | 0.663 | 0.648 | 0.602 |
|  | DFHiC | 0.741 | 0.760 | 0.796 | 0.831 | 0.819 | 0.789 |
| **QuASAR-Rep** | HiCPlus | 0.900 | 0.907 | 0.914 | 0.913 | 0.923 | 0.911 |
|  | SRHiC | 0.914 | 0.919 | 0.931 | 0.926 | 0.936 | 0.925 |
|  | HiCNN | 0.940 | 0.949 | 0.956 | 0.946 | 0.962 | 0.951 |
|  | **DeepHiC** | **0.948** | 0.949 | 0.958 | **0.956** | 0.963 | **0.955** |
|  | **HiCARN** | 0.947 | **0.951** | **0.959** | 0.954 | **0.964** | **0.955** |
|  | DFHiC | 0.936 | 0.950 | 0.956 | 0.943 | 0.963 | 0.950 |
| **HiCRep** | HiCPlus | 0.891 | 0.909 | 0.894 | 0.923 | 0.943 | 0.912 |
|  | SRHiC | 0.876 | 0.894 | 0.877 | 0.911 | 0.921 | 0.896 |
|  | HiCNN | 0.906 | 0.913 | 0.916 | 0.930 | 0.944 | 0.922 |
|  | DeepHiC | 0.888 | 0.878 | 0.881 | 0.908 | 0.902 | 0.891 |
|  | **HiCARN** | **0.916** | **0.923** | **0.922** | **0.946** | **0.945** | **0.930** |
|  | DFHiC | 0.893 | 0.902 | 0.902 | 0.915 | 0.931 | 0.909 |
| **HiC-Spetor** | HiCPlus | 0.723 | 0.283 | 0.650 | **0.733** | 0.745 | 0.627 |
|  | **SRHiC** | **0.760** | 0.236 | **0.793** | 0.711 | **0.795** | **0.659** |
|  | HiCNN | 0.398 | **0.316** | 0.419 | 0.412 | 0.388 | 0.387 |
|  | DeepHiC | 0.283 | 0.223 | 0.266 | 0.183 | 0.270 | 0.245 |
|  | HiCARN | 0.214 | 0.290 | 0.302 | 0.360 | 0.342 | 0.302 |
|  | DFHiC | 0.334 | 0.304 | 0.525 | 0.440 | 0.393 | 0.399 |

**Table S25. The Reproducibility Scores of all enhanced Hi-C matrices and high-resolution Hi-C matrices on chromosomes 18-22 in NHEK.**

|  | **Model** | **Chr18** | **Chr19** | **Chr20** | **Chr21** | **Chr22** | **Avg** |
| --- | --- | --- | --- | --- | --- | --- | --- |
| **GenomeDISCO** | **HiCPlus** | **0.821** | **0.777** | **0.802** | **0.813** | **0.797** | **0.802** |
|  | SRHiC | 0.808 | 0.755 | 0.789 | 0.801 | 0.779 | 0.786 |
|  | HiCNN | 0.743 | 0.710 | 0.722 | 0.717 | 0.717 | 0.722 |
|  | DeepHiC | 0.146 | -0.013 | 0.027 | -0.206 | -0.249 | -0.059 |
|  | HiCARN | 0.254 | 0.339 | 0.340 | 0.440 | 0.378 | 0.350 |
|  | DFHiC | 0.673 | 0.656 | 0.670 | 0.715 | 0.682 | 0.679 |
| **QuASAR-Rep** | HiCPlus | 0.744 | 0.671 | 0.749 | 0.738 | 0.704 | 0.721 |
|  | SRHiC | 0.785 | 0.680 | 0.793 | 0.776 | 0.741 | 0.755 |
|  | HiCNN | 0.882 | 0.818 | 0.890 | 0.883 | 0.855 | 0.866 |
|  | DeepHiC | 0.897 | 0.813 | 0.896 | 0.897 | 0.857 | 0.872 |
|  | **HiCARN** | **0.904** | **0.840** | **0.910** | **0.905** | **0.879** | **0.888** |
|  | DFHiC | 0.890 | 0.828 | 0.899 | 0.893 | 0.868 | 0.876 |
| **HiCRep** | HiCPlus | 0.813 | 0.841 | 0.793 | 0.801 | 0.868 | 0.823 |
|  | SRHiC | 0.784 | 0.828 | 0.779 | 0.791 | 0.839 | 0.804 |
|  | HiCNN | 0.820 | 0.828 | **0.820** | **0.832** | 0.862 | 0.832 |
|  | DeepHiC | 0.777 | 0.706 | 0.723 | 0.727 | 0.739 | 0.734 |
|  | **HiCARN** | **0.846** | **0.875** | 0.793 | 0.834 | **0.885** | **0.847** |
|  | DFHiC | 0.820 | 0.859 | 0.765 | 0.808 | 0.850 | 0.820 |
| **HiC-Spetor** | **HiCPlus** | **0.792** | 0.354 | 0.740 | **0.487** | **0.675** | **0.610** |
|  | SRHiC | 0.738 | **0.376** | **0.780** | 0.407 | 0.632 | 0.587 |
|  | HiCNN | 0.660 | 0.361 | 0.629 | 0.329 | 0.659 | 0.528 |
|  | DeepHiC | 0.269 | 0.197 | 0.258 | 0.125 | 0.101 | 0.190 |
|  | HiCARN | 0.217 | 0.230 | 0.364 | 0.324 | 0.408 | 0.309 |
|  | DFHiC | 0.434 | 0.344 | 0.434 | 0.374 | 0.663 | 0.450 |

**Table S26. The Reproducibility Scores of all enhanced Hi-C matrices and high-resolution Hi-C matrices on chromosomes 18-22 in CH12-LX.**

|  | **Model** | **Chr16** | **Chr17** | **Chr18** | **Chr19** | **Avg** |
| --- | --- | --- | --- | --- | --- | --- |
| **GenomeDISCO** | HiCPlus | 0.738 | 0.701 | 0.715 | 0.708 | 0.716 |
|  | SRHiC | 0.711 | 0.679 | 0.710 | **0.757** | 0.714 |
|  | **HiCNN** | **0.780** | **0.751** | **0.768** | 0.755 | **0.764** |
|  | DeepHiC | 0.268 | 0.068 | 0.266 | 0.184 | 0.197 |
|  | HiCARN | 0.209 | 0.265 | 0.167 | 0.294 | 0.234 |
|  | DFHiC | 0.643 | 0.636 | 0.639 | 0.660 | 0.645 |
| **QuASAR-Rep** | HiCPlus | 0.722 | 0.678 | 0.632 | 0.694 | 0.682 |
|  | SRHiC | 0.750 | 0.717 | 0.689 | 0.750 | 0.727 |
|  | HiCNN | 0.866 | 0.846 | 0.836 | 0.863 | 0.853 |
|  | DeepHiC | 0.847 | 0.812 | 0.820 | 0.858 | 0.834 |
|  | **HiCARN** | **0.876** | **0.852** | **0.851** | **0.879** | **0.865** |
|  | DFHiC | 0.856 | 0.835 | 0.826 | 0.862 | 0.845 |
| **HiCRep** | HiCPlus | 0.816 | 0.812 | 0.736 | 0.743 | 0.777 |
|  | SRHiC | 0.812 | 0.799 | 0.731 | 0.732 | 0.769 |
|  | **HiCNN** | 0.850 | **0.843** | **0.778** | **0.787** | **0.815** |
|  | DeepHiC | 0.764 | 0.691 | 0.663 | 0.684 | 0.701 |
|  | HiCARN | **0.862** | 0.816 | 0.771 | 0.760 | 0.802 |
|  | DFHiC | 0.832 | 0.808 | 0.734 | 0.727 | 0.775 |
| **HiC-Spetor** | HiCPlus | 0.543 | 0.441 | 0.584 | 0.429 | 0.499 |
|  | **SRHiC** | **0.607** | **0.654** | **0.598** | **0.711** | **0.643** |
|  | HiCNN | 0.452 | 0.440 | 0.524 | 0.434 | 0.463 |
|  | DeepHiC | 0.184 | 0.155 | 0.183 | 0.118 | 0.160 |
|  | HiCARN | 0.233 | 0.217 | 0.293 | 0.161 | 0.226 |
|  | DFHiC | 0.375 | 0.373 | 0.385 | 0.289 | 0.356 |

**Table S27. The comparison results of all enhancement methods on the test set for low-resolution Hi-C samples downsampled under different ratios (1/50, 1/100).**

|  | **Model** | **MSE** | **PSNR** | **SSIM** |
| --- | --- | --- | --- | --- |
| **Ratio 1:50** | HiCPlus | 48.299 | 13.407 | 0.125 |
|  | SRHiC | 45.146 | 13.972 | 0.037 |
|  | HiCNN | 15.097 | 18.326 | 0.146 |
|  | DeepHiC | 15.181 | 18.163 | 0.196 |
|  | HiCARN | 13.224 | 18.733 | 0.187 |
|  | **DFHiC** | **13.061** | **18.937** | **0.233** |
| **Ratio 1:100** | HiCPlus | 41.93 | 13.868 | 0.089 |
|  | SRHiC | 58.499 | 12.721 | 0.012 |
|  | HiCNN | 18.196 | 17.466 | 0.106 |
|  | DeepHiC | 16.914 | 17.842 | 0.129 |
|  | HiCARN | 15.197 | 18.206 | 0.121 |
|  | **DFHiC** | **15.03** | **18.224** | **0.156** |
